# Synthesis, Cytotoxicity, and Photophysical Investigations of 2-Amino-4,6-diphenylnicotinonitriles: An Experimental and Theoretical Study

**DOI:** 10.3390/molecules29081808

**Published:** 2024-04-16

**Authors:** Alwah R. Al-Ghamdi, Shofiur Rahman, Reem I. Al-Wabli, Maha S. Al-Mutairi, A. F. M. Motiur Rahman

**Affiliations:** 1 Department of Pharmaceutical Chemistry, College of Pharmacy, King Saud University, Riyadh 11451, Saudi Arabia; 439204477@student.ksu.edu.sa (A.R.A.-G.); ralwabli@ksu.edu.sa (R.I.A.-W.); malmutbiri@ksu.edu.sa (M.S.A.-M.); 2 College of Science, King Saud University, Riyadh 11451, Saudi Arabia; mrahman1@ksu.edu.sa

**Keywords:** 2-amino-4,6-diphenylnicotinonitrile, cytotoxicity, photophysical properties, DFT, TD-DFT

## Abstract

In this study, we present a comprehensive investigation of 2-amino-4,6-diphenylnicotinonitriles (APNs, **1**–**6**), including their synthesis, cytotoxicity against breast cancer cell lines, and photophysical properties. Compound **3** demonstrates exceptional cytotoxicity, surpassing the potency of Doxorubicin. The fluorescence spectra of the synthesized **1**–**6** in different solvents reveal solvent-dependent shifts in the emission maximum values, highlighting the influence of the solvent environment on their fluorescence properties. A quantum chemical TD-DFT analysis provides insights into the electronic structure and fluorescence behavior of **1**–**6**, elucidating HOMO-LUMO energy gaps, electronegativity values, and dipole moments, contributing to a deeper understanding of their electronic properties and potential reactivity. These findings provide valuable knowledge for the development of APNs (**1**–**6**) as fluorescent sensors and potential anticancer agents.

## 1. Introduction

**Figure 1:**
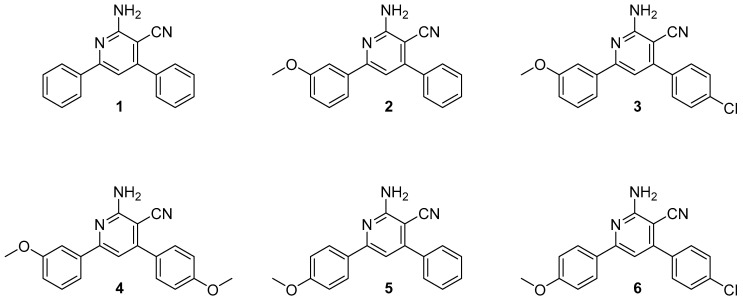
2-Amino-4,6-diphenylnicotinonitrile derivatives (**1**–**6**).

2-Amino-4,6-diphenylnicotinonitriles (APNs) are compounds of considerable interest due to their intriguing structure and diverse biological activities. APNs showed antimicrobial activity against various pathogens [1], antiproliferative activity with PIM-1 kinase inhibitory potential [2,3], anti-tubercular activity against Mycobacterium tuberculosis [4], SIRT1 inhibitory activity [5], and A_2A_ adenosine receptor antagonism [6]. In addition to their biological activities, APNs have also shown promise as fluorescent sensors for monitoring photopolymerization processes. APNs exhibited higher sensitivity compared to commercially available probes (7-diethylamino-4-methylcoumarin and trans-2-(2′,5′-dimethoxyphenyl)ethenyl-2,3,4,5,6-pentafluorobenzene) and have demonstrated their ability to accelerate cationic photopolymerization processes, particularly in the photoinitiation of epoxide and vinyl monomers [7]. This suggests the potential utility of APNs as fluorescent sensors for monitoring various polymerization processes and as long-wavelength co-initiators for diphenyliodonium salt initiators. Researchers have conducted extensive studies to synthesize these compounds using various approaches. Several research groups have successfully synthesized APNs from aldehydes and acetophenones with malonitrile, employing different reaction conditions. The synthesis of APNs has been achieved using solvent-free (neat) conditions, where the reaction takes place without the use of any additional solvents. This approach offers the advantages of simplicity and environmentally friendly characteristics [8,9,10]. Water has also been utilized as a reaction medium for synthesizing these compounds, providing a greener alternative [11,12,13,14]. Microwave irradiation [6,15,16,17] and ultrasonic irradiation [18,19] have also been employed as energy sources to expedite the reaction and enhance the yield and efficiency of the synthesis of APNs. Various catalysts have been employed to facilitate the synthesis of APNs. Notably, HBF_4_ has been used as an oxidizing promoter catalyst under mild and solvent-free conditions, providing an efficient and practical method for the synthesis of APNs [20]. Other catalysts include [HO_3_S-PhospIL@SBA-15] [21], the CoFe_2_O_4_-TRIS-sulfated boric acid nano catalyst [22], ClO_4_^−^/Al-MCM-41 nanoparticles [23], SrFe_12_O_19_ [24], amberlyst-15 [5], Cu(OAc)_2_ [1], nicotinium methane sulfonate (NMS) [17], doped nano-sized copper(I) oxide (Cu_2_O) on melamine–formaldehyde resin [25], [Bmim]BF_4_ ionic liquid [26], Bu_4_N^+^Br^−^ in aqueous medium [27], SnO_2_/SiO_2_ nanocomposite [28], ytterbium perfluorooctanoate [Yb(PFO)_3_] [29], poly *N*,*N*-dimethylaniline-formaldehyde supported on silica-coated magnetic nanoparticles [30], eggshell-based nano-magnetic solid acid catalyst [31], sulfonic acid-functionalized graphitic carbon nitride composite [32], and trifluoroethanol (TFE) [33]. Despite APNs’s intriguing structure and versatile biological activities, no comprehensive structural or photophysical studies have been conducted thus far. Therefore, in this study, we aim to address this research gap by presenting a convenient synthesis of APNs (Figure 1) and investigating their photophysical properties by examining the solvent effects on their emission spectra experimentally and computationally using density functional theory (DFT) and time-dependent density functional theory (TD-DFT). The DFT and TD-DFT computational methods were employed to provide insights concerning the physicochemical properties of these compounds at the molecular level. In light of the computational study and in good agreement with the experimental results, the resonance structures and the type of spectral bands in solution for the synthesized APN compounds were highlighted and discussed. This research will contribute to a deeper understanding of APNs’s characteristics and potential applications in various fields.

## 2. Results and Discussion

### 2.1. Synthesis of 2-Amino-4,6-diphenylnicotinonitriles (APNs, ***1***–***6***)

**Scheme 1:**
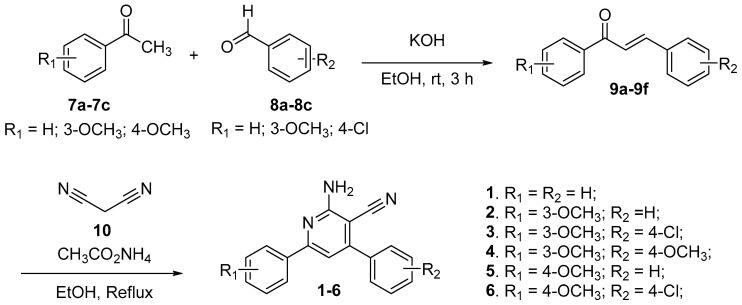
Synthesis of APNs (**1**–**6**).

The synthesis of the APNs (**1**–**6**) was efficiently achieved according to the depicted scheme (Scheme 1). The synthetic route involved a straightforward two-step process. In the initial step, a mixture of equimolar amounts of acetophenones (**7a**–**7c**) and aldehydes (**8a**–**8c**) was subjected to a reaction under basic conditions. This reaction yielded the corresponding chalcones [34]. Moving to the second step, the obtained chalcones (**9a**–**9f**) were then reacted with malononitrile (**10**) and ammonium acetate under refluxing conditions. This step involved heating the reaction mixture and allowing for efficient conversion and subsequent cyclization to form the desired APNs (**1**–**6**) [35]. The confirmation of the structures of the chalcone intermediates (**9a**–**9f**) and the final APNs (**1**–**6**) was accomplished through the utilization of various analytical techniques, including melting point determination, infrared (IR) spectroscopy, nuclear magnetic resonance (NMR) spectroscopy, and mass spectrometry. In addition to that, the observed melting points, IR spectra, NMR spectra, and mass spectra of known APNs (**1**–**6**) were compared with the expected values reported in the literature. These findings provide evidence for the successful synthesis of the desired APNs (**1**–**6**).

In brief, the infrared (IR) spectral analysis of **1**–**6** revealed characteristic absorption bands associated with the presence of specific functional groups, namely the amino (N-H) and cyanide (-CN) groups. The amino (N-H) group in **1**–**6** exhibited distinctive stretching vibrations in the IR spectra. The N-H stretching bands were observed in the range of 3412–3487 cm^−1^ and 3300–3368 cm^−1^. These absorption bands arise from the stretching motion of the nitrogen–hydrogen bonds in the amino group. The broad nature of these bands is typical for N-H stretching vibrations. Additionally, the N-H bending vibrations of the amino group were observed in the IR spectra of **1**–**6**. The N-H bending bands appeared in the range of 1606–1654 cm^−1^. These absorption bands result from the bending motion of the nitrogen–hydrogen bonds in the amino group. The presence of these bands further confirms the presence of the amino group in the synthesized compounds (**1**–**6**). Furthermore, the cyanide (-CN) groups in compounds **1**–**6** exhibited characteristic absorption bands in the IR spectra. The IR spectra showed strong peaks at around 2204–2210 cm^−1^, which correspond to the stretching vibrations of the carbon–nitrogen triple bond (-C≡N) in the cyanide group. These absorption bands are indicative of the presence of the cyanide group in compounds **1**–**6**. 

The proton nuclear magnetic resonance (^1^H-NMR) spectra of compounds **1**–**6** exhibit characteristic signals associated with specific proton environments, including the proton at the 5-position and the -NH_2_ group. In the ^1^H-NMR spectra, a characteristic singlet signal is observed at 7.09–7.25 ppm for the proton at the 5-position of compounds **1**–**6**. The -NH_2_ peaks of compounds **1**–**6** appear as a broad singlet at 5.30–5.38 ppm. The mass spectra of compounds **1**–**6** exhibit protonated molecular ions and, in some cases, sodium adducts. Additionally, compounds **3** and **6** show chlorine isotopes (^35^Cl and ^37^Cl) in their mass spectra.

### 2.2. Cytotoxicity of the Synthesized APNs (***1***–***6***)

The IC_50_ values of the synthesized APNs (**1**–**6**) and the control Doxorubicin were compared to assess their potency against the MDA-MB-231 and MCF-7 breast cancer cell lines (Table 1). Compound **3** emerged as the most promising compound, displaying excellent cytotoxicity with IC_50_ values of 1.81 ± 0.1 μM (MDA-MB-231) and 2.85 ± 0.1 μM (MCF-7). Notably, compound **3** exhibited higher potency than Doxorubicin, the control compound (Doxorubicin: IC_50_ = 3.18 ± 0.1 μM for MDA-MB-231 and 4.17 ± 0.2 μM for MCF-7). Compound **4** also demonstrated comparable activity to Doxorubicin, with IC_50_ values of 6.93 ± 0.4 μM (MDA-MB-231) and 5.59 ± 0.3 μM (MCF-7). Compound **2** displayed potent activity against both cell lines, albeit slightly lower than Doxorubicin (compound **2**: IC_50_ = 8.01 ± 0.5 μM for MDA-MB-231 and 16.20 ± 1.3 μM for MCF-7). Compounds **5** and **6** exhibited moderate potency against the tested cell lines (compound **5**: IC_50_ = 15.52 ± 1.2 μM for MDA-MB-231 and 20.07 ± 1.5 μM for MCF-7; compound **6**: IC_50_ = 10.23 ± 0.8 μM for MDA-MB-231 and 9.47 ± 0.7 μM for MCF-7). Compound **1** exhibited weak activity with an IC_50_ of 78.28 ± 3.9 μM against MDA-MB-231 cells, but it was not effective in inhibiting the growth of MCF-7 cells (IC_50_ > 100 μM). These findings indicate that compound **3** shows excellent cytotoxicity, surpassing the potency of Doxorubicin, while compound **4** is comparable to the control Doxorubicin. Further optimization and development of these compounds may lead to the discovery of highly potent and effective cytotoxic agents for breast cancer treatment. 

### 2.3. Photophysical Properties of APNs (***1***–***6***)

**Figure 2:**
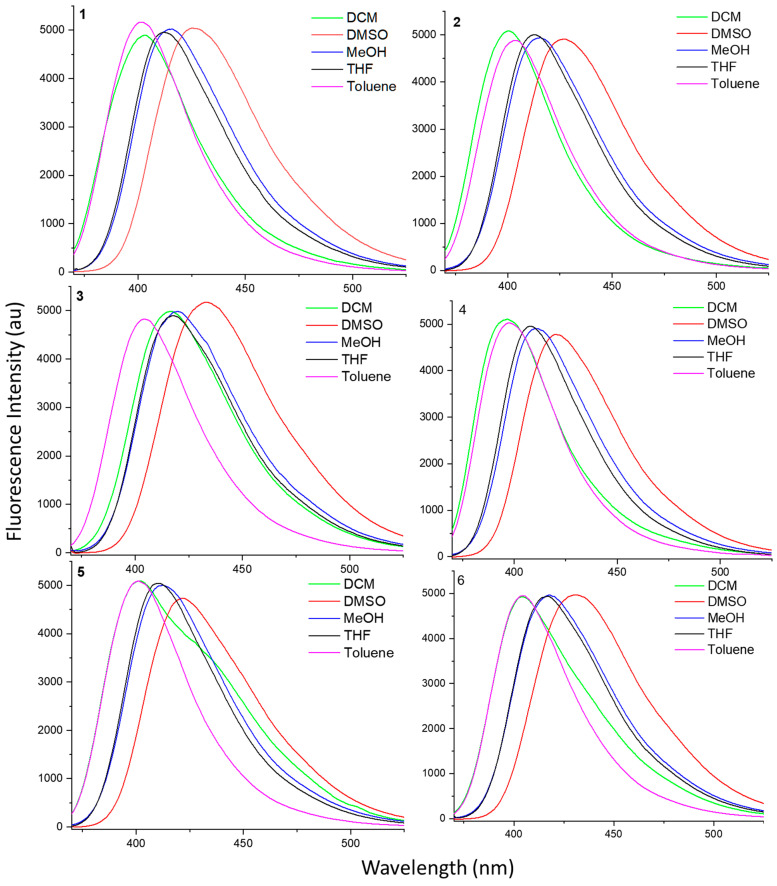
Fluorescence (emission) spectra of synthesized compounds **1**–**6** in different solvents at 1.45 × 10^−8^ M.

The fluorescence spectra of the synthesized APNs (**1**–**6**) in different solvents (DCM, DMSO, MeOH, THF, and Toluene) at a concentration of 1.45 × 10^−8^ M are shown in Figure 2. The fluorescence emission for each compound (**1**–**6**) is summarized in Table 2. As shown in Table 2, we observe different emission maximum values for each compound in different solvent systems. The varying λ_max_ values suggest that different solvents can influence the energy levels and electronic transitions involved in the fluorescence process. Solvents with different polarity and dielectric constants interacted differently with compounds **1**–**6**, leading to shifts in the emission maximum.

Several interesting observations can be made upon analyzing the emission maxima (λem) of compounds **1**–**6** in different solvents. As shown in Table 2, all six compounds exhibit similar trends in their fluorescence properties across the different solvents. This suggests that the core structure, compound **1**, significantly determines the overall fluorescence behavior. In all five solvents, all the compounds (**1**–**6**) give more or less similar fluorescence properties. A very little red shift is observed for polar solvents as well as electron-withdrawing/containing groups. When dissolved in dimethyl sulfoxide (DMSO), all compounds undergo a significant red shift compared to the other solvents. The emission maximum range is between 420 and 433 nm in DMSO, indicating a shift towards longer wavelengths. This red shift can be attributed to the polar nature of DMSO, which stabilizes charge-separated states and polar excited states, resulting in a longer-wavelength emission. Among the six compounds, compound **3** consistently exhibits higher emission maximum values in all solvents. This implies that compound **3** has a stronger ability to absorb light energy and undergo efficient electronic transitions, leading to enhanced fluorescence. The specific substituents, such as the chloro groups, present in compound **3** likely contribute to its favorable electronic properties. Compound **6** shows similar fluorescence behavior to compound **3**, as both compounds exhibit relatively higher emission maximum values compared to the other compounds. This suggests that the specific substitution of chloro groups in compound **6**, similar to compound **3**, contributes to its enhanced fluorescence properties. To summarize, the fluorescence properties of compounds **1**–**6** reveal intriguing patterns.

**Figure 3:**
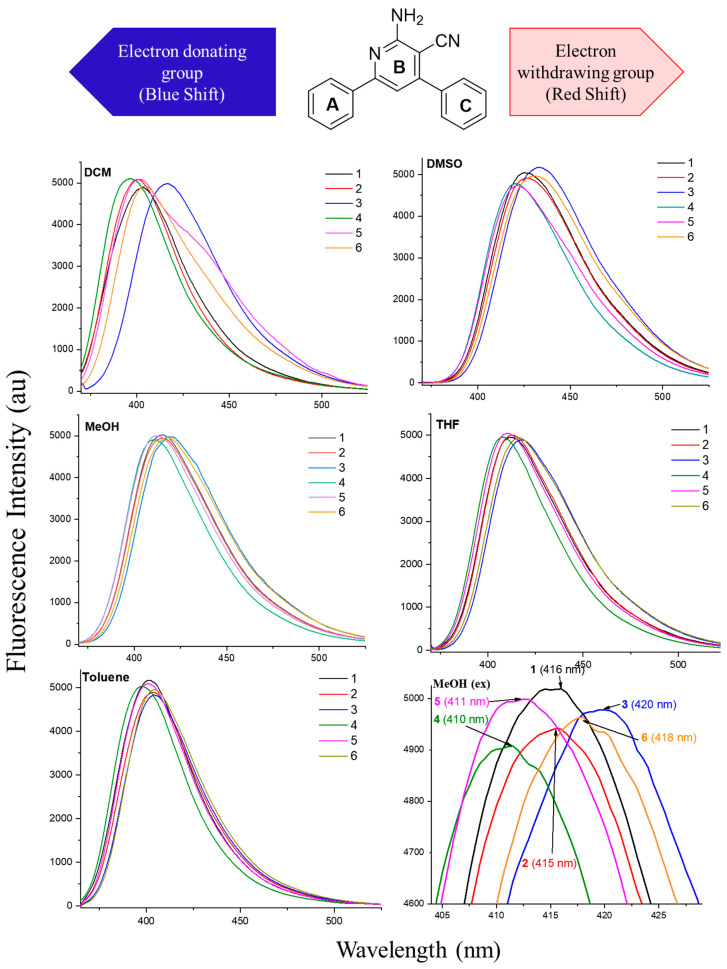
Electron-withdrawing/donating effect of fluorescence emission of compounds **1**–**6**.

While all the APNs (**1**–**6**) exhibit similarities in their fluorescence behavior, the choice of solvent has a significant influence on the emission maximum values (Figure 3). The solvents tetrahydrofuran (THF, 407–418 nm) and methanol (MeOH, 410–419 nm) display a pronounced red shift compared to dichloromethane (DCM, 396–416 nm) and toluene (397–405 nm). Additionally, dimethyl sulfoxide (DMSO, 420–433 nm) induces an exceptional red shift for all compounds. Compounds **3** and **6** consistently exhibit higher emission maximum values across all solvents, indicating their superior fluorescence properties. These findings provide valuable insights into the structure–property relationships and solvatochromic effects in the fluorescence of these APNs (**1**–**6**). Besides the solvent effect, there is clear evidence of electron with/donating group effects on emission shifting. Figure 3 illustrates the fluorescence properties of the compounds in methanol (extension). Compound **1**, without any substituents, exhibits an emission maximum at 416 nm. Substitutions of a 3-methoxy group in ring A (compound **2**), a 4-methoxy group in ring A (compound **5**), and both a 3-methoxy group in ring A and a 4-methoxy group in ring B (compound **4**) result in blue shifts of approximately **1**–**6** nm, with emission maxima at 415, 411, and 410 nm, respectively. Conversely, compound **6**, with a 3-methoxy group in ring A and a 4-chloro group in ring B, and compound **3**, with a 4-methoxy group in ring A and a 4-chloro group in ring B, exhibit red shifts of approximately 2–4 nm, with emission maxima at 418 and 420 nm, respectively. These observations highlight the influence of electron with/donating substitutions on the fluorescence properties of the APNs (**1**–**6**). The red and blue shifts observed in the APNs (**1**–**6**) have diverse applications. These shifts can be utilized to develop fluorescent probes for imaging and sensing applications, where the emission wavelengths can be tuned for specific targets. Compounds **1**–**6** can also be used in optical sensors to detect analytes or environmental parameters based on changes in emission spectra. Additionally, the red and blue shifts are valuable in energy transfer systems and can be applied in areas such as solar energy conversion and light harvesting. Overall, the red and blue shifts of the APNs (**1**–**6**) offer versatile applications in various fields, ranging from biological imaging to energy-related technologies. However, the analysis of compounds **1**–**6** reveals several significant findings for the optimization and development of APN therapeutics or fluorescent sensors. The choice of solvent influences the emission maximum values, suggesting the importance of solvent selection for tuning fluorescence properties. Specific substituents, such as chloro groups, contribute to enhanced fluorescence properties, as observed in compounds **3** and **6**. Additionally, the position of methoxy groups (3-OCH_3_ vs. 4-OCH_3_) affects the emission maxima, indicating their role in determining donor/acceptor sites. These findings offer valuable insights for designing APN-based compounds with improved fluorescence behavior.

### 2.4. Quantum Chemical TD-DFT Analysis of APNs (***1***–***6***)

**Figure 4:**
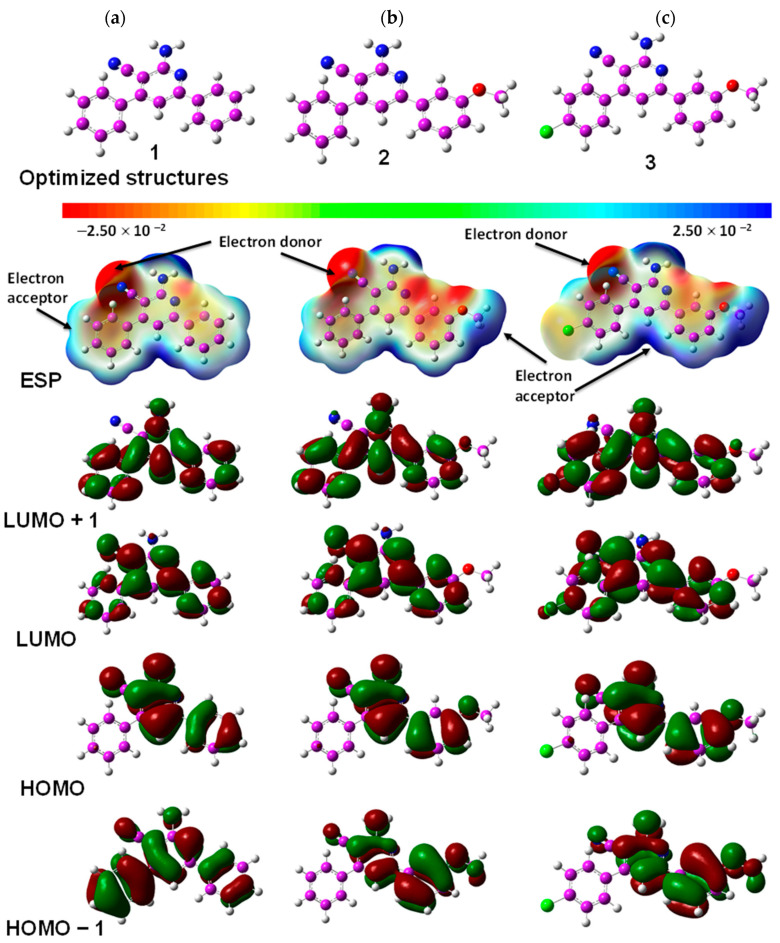
Optimized gas-phase molecular structures, ESP, LUMO, and HOMO structures of APNs **1**–**3** ((**a**) compound **1**; (**b**) compound **2**; and (**c**) compound **3**) at the B3LYP/6-311++g(d,p) level of theory in the water solvent system (color code: carbon: purple; hydrogen: white; nitrogen: blue; oxygen: red; sulfur: yellow). The order of increasing ESP (i.e., highest negative value) is red > orange > yellow > green > blue (indicated by the arrows).

**Figure 5:**
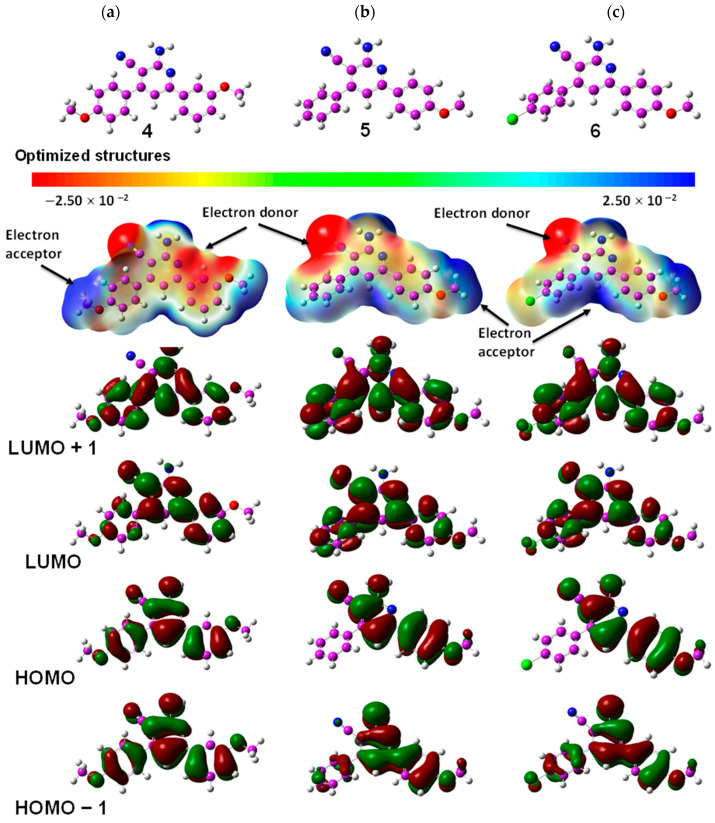
Optimized gas-phase molecular structures, ESP, LUMO, and HOMO structures of APNs **4**–**6**: ((**a**) compound **4**; (**b**) compound **5**; and (**c**) compound **6**) at the B3LYP/6-311++g(d,p) level of theory in the water solvent system (color code: carbon: purple; hydrogen: white; nitrogen: blue; oxygen: red; sulfur: yellow). The order of increasing ESP (i.e., highest negative value) is red > orange > yellow > green > blue (indicated by the arrows).

In order to gain a deep insight into the structure of the synthesized compounds **1**–**6**, a time-dependent density functional theory (TD-DFT) approach was conducted to model the electronic fluorescence spectra of the synthesized APNs (**1**–**6**) and compare them with the experimental ones. A chemical species with a small HOMO-LUMO energy gap is highly polarizable and requires only a small amount of energy for excitation. On the other hand, a large HOMO-LUMO gap indicates that the species is highly stable and has low reactivity. The molecular electrostatic surface potentials, HOMO, and LUMO of the APNs (**1**–**6**) are shown in Figure 4 and Figure 5, and the important HOMO, LUMO, and their related values are summarized in Table 3. The negative electrostatic potential (ESP) of the species is shown in red, indicating the relative polarities and reactive sites. The order of increasing electrostatic potential (i.e., the highest negative value) is red > orange > yellow > green > blue. The yellow color indicates the slightly rich electron regions, and the green color reflects more neutral zones. The HOMO–LUMO transition implies an electron density transfer from the ring to the lone pairs of atoms on the main chain. This explains the significant degree of intramolecular charge transfer by the π–π and n–π transitions.

The results obtained from Table 3 provide valuable insights into the electronic properties of the compounds, specifically the HOMO-LUMO energy levels and the electronegativity (χ) values. The HOMO-LUMO energy gap is a crucial parameter that reflects the electronic structure and potential reactivity of compounds. In this study, all the compounds displayed comparable HOMO-LUMO energy gaps, ranging from 3.441 eV to 3.617 eV. This indicates a consistent degree of electronic delocalization and suggests that these compounds may exhibit similar reactivity patterns. The property of attracting electrons, known as electronegativity (χ), serves as a gauge for predicting chemical reactivity. The electronegativity values, ranging from 4.235 eV to 4.435 eV, highlight the varying electron-attracting capabilities among atoms in the synthesized APNs (**1**–**6**). These values offer a deeper understanding of the compounds’ electronic characteristics and their propensity for electron transfer reactions. The similarity in the HOMO-LUMO energy gaps and the range of electronegativity values observed in the compounds (**1**–**6**) suggest that they may share comparable electronic properties. This information is valuable for understanding their potential reactivity and applications. The chemical potential (μ) for an electron in a molecule represents its ability to be either added to or removed from the molecule, and it is a critical parameter in understanding the electronic behavior and reactivity of chemical systems. The chemical potential (μ) values, ranging from −4.235 eV to −4.435 eV, highlight the varying electron-attracting capabilities among atoms in compounds **1**–**6**. The dipole moment of a molecule can significantly influence its emission spectra. When a molecule moves from a higher to a lower energy state, such as in fluorescence or phosphorescence, the dipole moment is crucial in determining the intensity and shape of the emitted spectrum. Additionally, solvent polarity can modulate a molecule’s dipole moment, leading to changes in the emission spectra. It can be seen that the dipole moment values are higher for compounds **3**, **5**, and **6** (11.787, 6.726, and 8.098, respectively). These values influence their emission spectra to undergo a red shift at higher wavelengths in the DMSO solvent (309/414, 316/420, and 345/427 nm, respectively) compared to other less polar solvents, as shown in Table 4.

The TD-DFT analysis of compounds **1**–**6** provides valuable insights for the development of APN therapeutics or fluorescent sensors. The similar HOMO-LUMO energy gaps and range of electronegativity values suggest comparable electronic properties among the compounds. The chemical potential values indicate varying electron-attracting capabilities. The higher dipole moment values of compounds **3**, **5**, and **6** suggest the potential for red-shifted emission spectra in polar solvents. Additionally, the position of methoxy groups influences donor/acceptor sites. These findings aid in optimizing the APN compounds (**1**–**6**) for the desired properties and applications.

**Figure 6:**
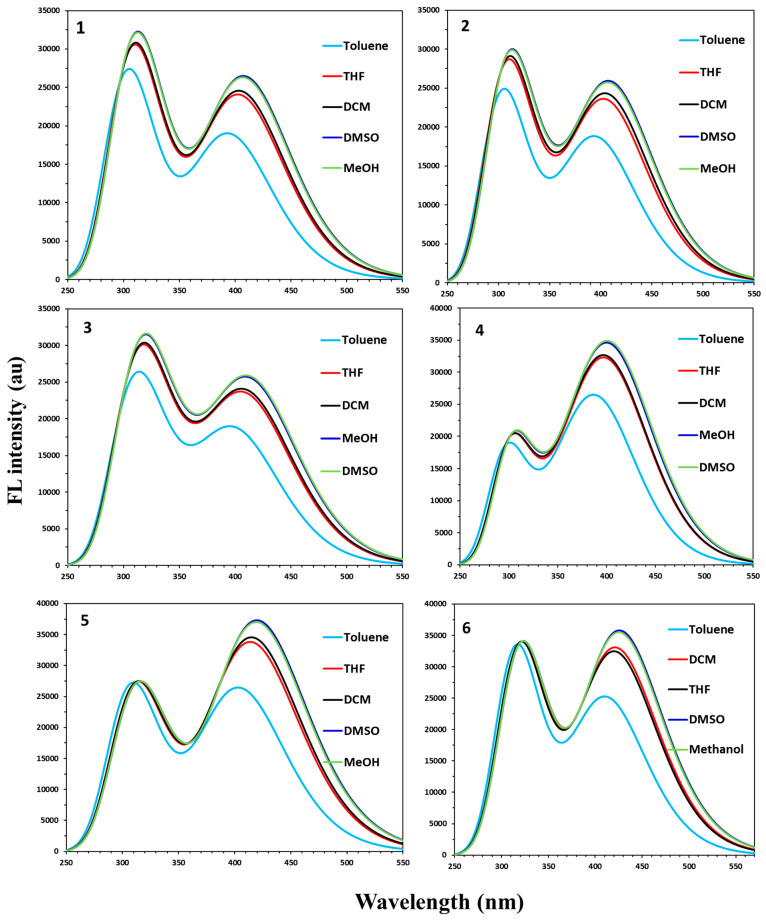
TD-DFT-calculated electronic fluorescence emission spectra of synthesized APNs (**1**–**6**) at the TD-DFT/B3LYP/6-311++g(d,p) basis set level of theory in toluene, THF, DCM, DMSO, and methanol solvent systems.

**Figure 7:**
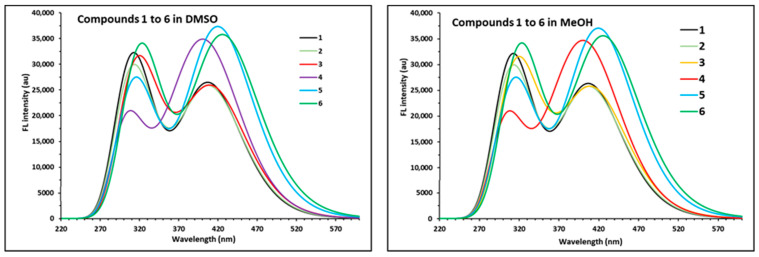
Comparison of the TD-DFT-calculated electronic fluorescence emission spectra of compounds **1**–**6** at the TD-DFT/B3LYP/6-311++g(d,p) basis set level of theory in DMSO and methanol solvent systems.

The geometry-optimized structures of APNs **1**–**6** were further TD-DFT-calculated by computing five singlet excited states on top of the structures obtained at the TD-DFT/b3lyp/6-311++g(d,p) level of theory with the solvent polarizable continuum model (PCM) in toluene, tetrahydrofuran (THF), dichloromethane (DCM), dimethyl sulfoxide (DMSO), and methanol solvents, as shown in Figure 6 and Figure 7.

**Figure 8:**
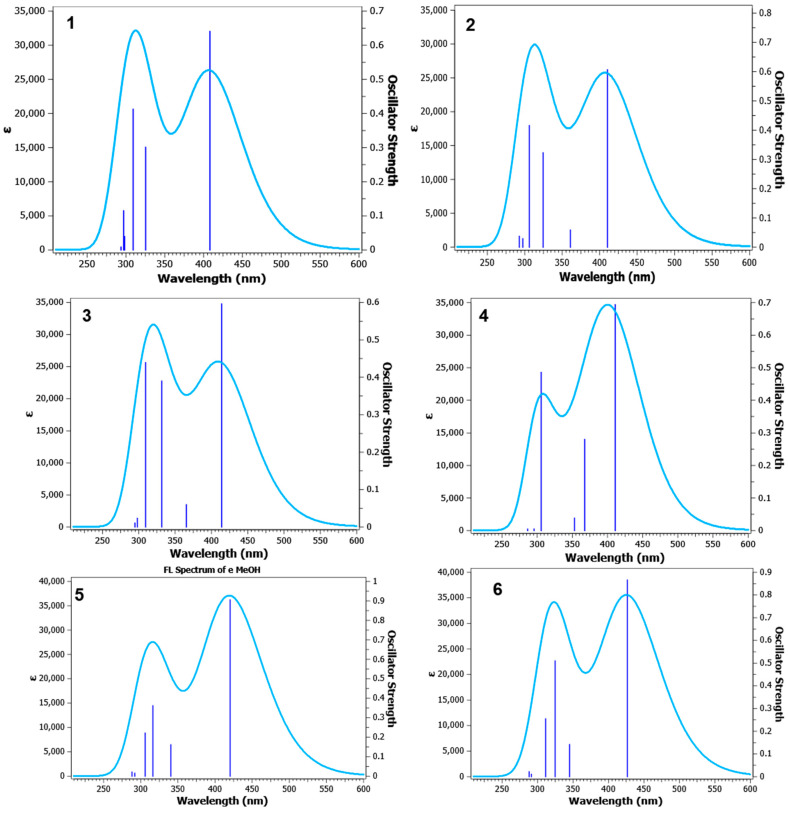
TD-DFT-calculated electronic fluorescence emission spectra of compounds **1**–**6** at the TD-DFT/B3LYP/6-311++g(d,p) basis set level of theory in a methanol solvent system.

**Figure 9:**
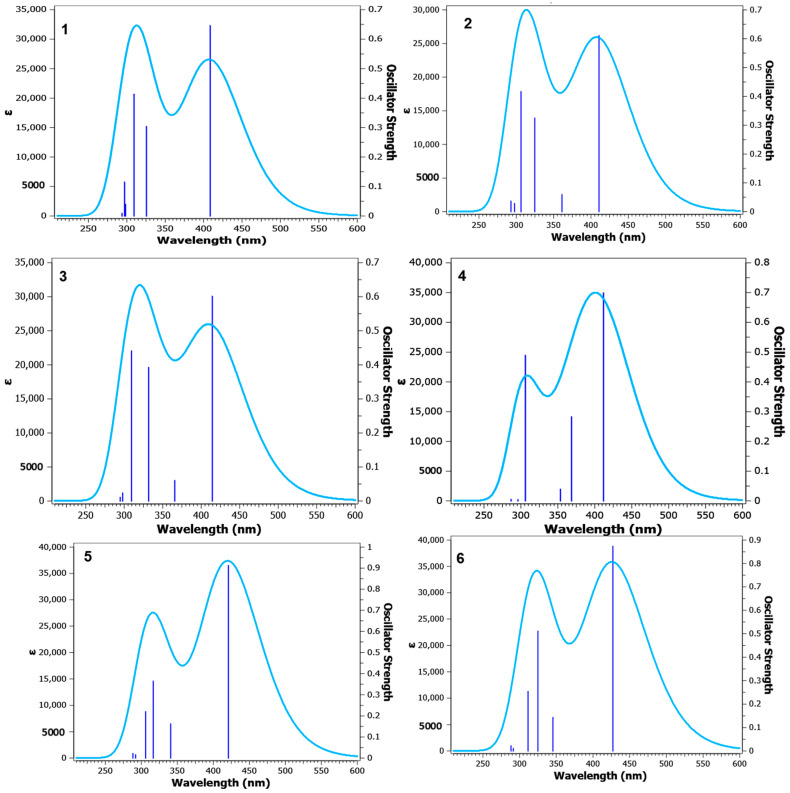
TD-DFT-calculated electronic fluorescence emission spectra of compounds **1**–**6** at the TD-DFT/B3LYP/6-311++g(d,p) basis set level of theory in a DMSO solvent system.

The spectra were plotted by applying a Gaussian broadening of 0.333 eV half-width at half-height. The fluorescence emission spectra exhibit two bands of compounds **1**–**6** in toluene, THF, DCM, DMSO, and methanol solvents. These bands are at 299−325 nm and 394−427 nm, respectively, as summarized in Table 4. The short-wavelength region in 299−325 nm is assigned to the n–π* electron transition of the periphery of the APN moieties. The region 394−427 nm is consistent with the π−π* electron transition due to charge transfer emission (Figure 8 and Figure 9).

As depicted, the TD-DFT-calculated electronic fluorescence emission spectra of compounds **1**–**6** were obtained at the TD-DFT/B3LYP/6-311++g(d,p) basis set level of theory in the MeOH (Figure 8) and DMSO (Figure 9) solvent systems at 298 K. For compound **1**, Figure 8 shows two broad bands at 408.18 nm (excitation energy of 3.037 eV and oscillator strength f = 0.640 for HOMO to LUMO) and 309 nm (excitation energy of 4.009 eV and oscillator strength f = 0.412). Similarly, in Figure 9, compound **1** displays two broad bands at 408.14 nm (excitation energy of 3.037 eV and oscillator strength f = 0.640 for HOMO to LUMO) and 309 nm (excitation energy of 4.009 eV and oscillator strength f = 0.412 for HOMO-2 to LUMO). For compound **2**, Figure 8 shows two broad bands at 410.30 nm (excitation energy of 3.022 eV and oscillator strength f = 0.606 for HOMO to LUMO) and 306 nm (excitation energy of 4.050 eV and oscillator strength f = 0.415 for HOMO-4 to LUMO). Similarly, in Figure 9, compound **2** exhibits two broad bands at 410.72 nm (excitation energy of 3.018 eV and oscillator strength f = 0.610 for HOMO to LUMO) and 306.23 nm (excitation energy of 4.048 eV and oscillator strength f = 0.416 for HOMO-4 to LUMO). For compound **3**, Figure 8 shows two broad bands at 414.07 nm (excitation energy of 2.994 eV and oscillator strength f = 0.596 for HOMO to LUMO) and 309.38 nm (excitation energy of 4.007 eV and oscillator strength f = 0.439 for HOMO-3 to LUMO). Similarly, in Figure 9, compound **3** exhibits two broad bands at 410.47 nm (excitation energy of 2.991 eV and oscillator strength f = 0.601 for HOMO to LUMO) and 309.54 nm (excitation energy of 4.005 eV and oscillator strength f = 0.440 for HOMO-3 to LUMO). For compound **4**, Figure 8 shows two broad bands at 411.20 nm (excitation energy of 3.015 eV and oscillator strength f = 0.694 for HOMO to LUMO) and 305.81 nm (excitation energy of 4.007 eV and oscillator strength f = 0.486 for HOMO-3 to LUMO). Similarly, in Figure 9, compound **4** exhibits two broad bands at 411.68 nm (excitation energy of 3.011 eV and oscillator strength f = 0.698 for HOMO to LUMO) and 306.02 nm (excitation energy of 4.051 eV and oscillator strength f = 0.487 for HOMO-3 to LUMO). For compound **5**, Figure 8 displays two broad bands at 420.37 nm (excitation energy of 2.949 eV and oscillator strength f = 0.905 for HOMO to LUMO) and 316.38 nm (excitation energy of 3.918 eV and oscillator strength f = 0.362 for HOMO-2 to LUMO). Similarly, in Figure 9, compound **5** exhibits two broad bands at 420.88 nm (excitation energy of 2.945 eV and oscillator strength f = 0.912 for HOMO to LUMO) and 316.52 nm (excitation energy of 3.917 eV and oscillator strength f = 0.363 for HOMO-2 to LUMO). For compound **6**, Figure 8 shows two broad bands at 426.66 nm (excitation energy of 2.905 eV and oscillator strength f = 0.866 for HOMO to LUMO) and 324.79 nm (excitation energy of 3.817 eV and oscillator strength f = 0.509 for HOMO-2 to LUMO). Similarly, in Figure 9, compound **6** exhibits two broad bands at 427.16 nm (excitation energy of 2.902 eV and oscillator strength f = 0.873 for HOMO to LUMO) and 324.90 nm (excitation energy of 3.816 eV and oscillator strength f = 0.510 for HOMO-2 to LUMO). The emission bands experienced a red shift when the solvent was changed from non-polar toluene to polar DMSO or a methanol solvent system. The highest red shift was observed for compounds **3**, **5**, and **6** from 304/401 to 309/414, 311/404 to 316/420, and 320/412 to 325/427, respectively.

## 3. Materials and Methods 

### 3.1. General

The experiments were conducted using commercially available reagents and solvents without any additional purification. Melting points were measured using a Barnstead electrothermal digital melting point apparatus (model IA9100, BIBBY Scientific Limited, Staffordshire, UK). IR spectra were recorded using a Jasco FT/IR-6600 spectrometer (Tokyo, Japan). NMR spectra were obtained using a Bruker 700 MHz NMR spectrometry instrument (Zurich, Switzerland); ^1^H NMR, ^13^C NMR, and 2D spectra were determined on a Bruker (700 MHz). Chemical shifts are expressed as δ values (ppm) using tetramethyl silane (TMS) as an internal reference. Signals are indicated by the following abbreviations: s = singlet, d = doublet, t = triplet, q = quartet, m = multiple, br = broad, dd = doublet of doublet, ddd = doublet of doublet of doublet, td = triplet of doublet, dt = doublet of triplet, and qd = quartet of doublet. Electrospray ionization (ESI) mass spectrometry (MS) experiments were carried out using an Agilent 6320 ion trap mass spectrometer equipped with an ESI ion source (Agilent Technologies, Palo Alto, CA, USA). High-resolution mass spectrometry was performed using an HRMS JMS 700 JEOL mass spectrometry instrument (Tokyo, Japan). The reagents were RPMI-1640 medium, MTT, and DMSO (Sigma Co., St. Louis, MO, USA), as well as fetal bovine serum (GIBCO, Cambridge, UK). The cell lines (MDA-MB-231, association number HTB-26^™^; and MCF-7, association number HTB-22) were obtained from the ATCC database via the Holding company for biological products and vaccines (VACSERA), Cairo, Egypt. 

### 3.2. General Synthetic Method for the Synthesis of ***1***–***6***

Compounds **1**–**6** were synthesized according to the previously reported method [35]. In the first step, chalcones (**9**) were synthesized using various aldehydes (**7**, 10 mmol) and acetophenones (**8**, 10 mmol) under a basic (10% alcoholic NaOH) condition in ethanol [34] at room temperature and were used for the second step without further purification. In the second step, each chalcone (1 mmol) was reacted with 1 equivalent of malononitrile and ammonium acetate (3 equiv.) under reflux overnight in absolute ethanol to obtain 2-amino-4,6-diphenylnicotinonitrile derivatives **1**–**6**.

#### 3.2.1. 2-Amino-4,6-diphenylnicotinonitrile (**1**)

Yellow powder (92%). Mp. 189 °C (lit. mp. 187–188 °C [35]; 179–181 °C [36]). FT-IR (KBr): ν (cm^−1^) = 3463, 3299, 3170, 2205, 1637, 1572, 1548, 1495, 1451, 1369, 1257, 1075, 848, 756, 699, and 525 cm^−1^. ^1^H-NMR (700 MHz, CDCl_3_) δ 8.03 (dd, *J* = 7.9, 1.8 Hz, 2H), 7.67 (dd, *J* = 7.9, 1.7 Hz, 2H), 7.55 (q, *J* = 7.7, 7.0 Hz, 3H), 7.52–7.49 (m, 3H), 7.25 (s, 1H), and 5.38 (s, 2H) ppm. ^13^C-NMR (176 MHz, CDCl_3_) δ 160.23, 159.85, 155.15, 137.96, 136.95, 130.22, 129.85, 128.97, 128.83, 128.19, 127.34, 117.15, 111.32, and 88.33 ppm. Mass (ESI): *m/z* 271.9693 [M + H]^+^; 293.9424 [M + Na]^+^.

#### 3.2.2. 2-Amino-6-(3-methoxyphenyl)-4-phenylnicotinonitrile (**2**)

White powder (76.4%). Mp. 163 °C (lit. mp. 163–164 °C [37]). FT-IR (KBr): ν (cm^−1^) = 3417, 3322, 3202, 3019, 2210, 1651, 1569, 1556, 1494, 1288, 1258, 1051, 904, 754, 779, 523, and 478 cm^−1^. ^1^H-NMR (700 MHz, CDCl_3_) δ 7.62 (d, *J* = 5.9 Hz, 2H), 7.56 (s, 1H), 7.54 (d, *J* = 7.7 Hz, 1H), 7.50 (d, *J* = 8.3 Hz, 3H), 7.36 (t, *J* = 7.9 Hz, 1H), 7.18 (s, 1H), 6.99 (d, *J* = 8.0 Hz, 1H), 5.33 (s, 2H), and 3.87 (s, 3H) ppm. ^13^C-NMR (176 MHz, CDCl_3_) δ 161.60, 160.19, 159.57, 153.63, 136.04, 135.50, 130.17, 129.53, 129.23, 128.88, 117.15, 114.22, 110.19, 87.14, and 55.45 ppm. Mass (ESI): *m/z* 302.0671 [M + H]^+^; 324.0705 [M + Na]^+^.

#### 3.2.3. 2-Amino-4-(4-chlorophenyl)-6-(3-methoxyphenyl)nicotinonitrile (**3**)

White powder (80.6%). Mp. 195 °C. FT-IR (KBr): ν (cm^−1^) = 3412, 3327, 3211, 2946, 2836, 2209, 1652, 1571, 1552, 1494, 1289, 1256, 1093, 1053, 1015, 844, 816, 815, 768, 523, and 488 cm^−1^. ^1^H-NMR (700 MHz, CDCl_3_) δ 7.55 (d, *J* = 8.3 Hz, 3H), 7.53 (d, *J* = 7.7 Hz, 1H), 7.49 (d, *J* = 6.6 Hz, 2H), 7.37 (t, *J* = 7.8 Hz, 1H), 7.14 (s, 1H), 7.00 (d, *J* = 8.0 Hz, 1H), 5.34 (s, 2H), and 3.87 (s, 3H) ppm. ^13^C-NMR (176 MHz, CDCl_3_) δ 160.16, 160.07, 159.84, 153.83, 139.21, 136.18, 135.29, 129.86, 129.54, 129.27, 119.71, 116.90, 116.20, 112.66, 111.16, 88.15, 55.45, and 55.45 ppm. Mass (ESI): *m/z* 336.0031 [M(^35^Cl) + H]^+^; 338.0375 [M(^37^Cl) + H]^+^.

#### 3.2.4. 2-Amino-6-(3-methoxyphenyl)-4-(4-methoxyphenyl)nicotinonitrile (**4**)

Green powder (72.5%). Mp. 165 °C. FT-IR (KBr): ν (cm^−1^) = 3447, 3414, 3329, 3216, 2938, 2835, 2208, 1654, 1643, 1612, 1516, 1295, 1254, 1178, 1052, 1034, 816, 767, and 505 cm^−1^. ^1^H-NMR (700 MHz, CDCl_3_) δ 7.59 (d, *J* = 8.8 Hz, 2H), 7.55 (s, 1H), 7.53 (d, *J* = 7.9 Hz, 1H), 7.36 (t, *J* = 7.9 Hz, 1H), 7.16 (s, 1H), 7.02 (d, *J* = 7.3 Hz, 2H), 6.98 (d, *J* = 8.1 Hz, 1H), 5.30 (s, 2H), and 3.86 (s, 6H) ppm. ^13^C-NMR (176 MHz, CDCl_3_) δ 161.01, 160.26, 160.04, 159.48, 154.72, 139.55, 129.80, 129.65, 129.16, 119.71, 117.47, 116.03, 114.40, 112.59, 111.21, 88.14, 55.45, and 55.44 ppm. Mass (ESI): *m/z* 332.1401 [M + H]^+^; 354.0757 [M + Na]^+^; 370.0165 [M + K]^+^.

#### 3.2.5. 2-Amino-6-(4-methoxyphenyl)-4-phenylnicotinonitrile (**5**)

Yellow powder (66.4%). Mp. 178 °C (lit. mp. 176–178 °C [38]). FT-IR (KBr): ν (cm^−1^) = 3487, 3368, 3200, 3066, 2970, 2204, 1897, 1617, 1606, 1572, 1543, 1238, 1172, 1027, 829, 766, 700, and 516 cm^−1^. ^1^H-NMR (700 MHz, CDCl_3_) δ 8.02 (d, *J* = 8.8 Hz, 2H), 7.66 (d, *J* = 6.9 Hz, 2H), 7.57–7.51 (m, 3H), 7.19 (s, 1H), 7.03–7.00 (m, 2H), 5.34 (s, 2H), and 3.90 (s, 4H) ppm. ^13^C-NMR (176 MHz, CDCl_3_) δ 161.49, 160.19, 159.34, 154.94, 137.11, 130.36, 129.76, 128.93, 128.86, 128.17, 117.35, 114.18, 110.49, 87.45, and 55.44 ppm. Mass (ESI): *m/z* 302.0650 [M + H]^+^; 324.0657 [M + Na]^+^.

#### 3.2.6. 2-Amino-4-(4-chlorophenyl)-6-(4-methoxyphenyl)nicotinonitrile (**6**)

Pink powder (90.1%). Mp. 195 °C (lit. mp. 194–197 °C [38]). FT-IR (KBr): ν (cm^−1^) = 3458, 3367, 3230, 2833, 2207, 1638, 1607, 1575, 1546, 1493, 1235, 1173, 1091, 1013, 822, 808, and 495 cm^−1^. ^1^H-NMR (700 MHz, CDCl_3_) δ 7.96 (d, *J* = 8.5 Hz, 2H), 7.55 (d, *J* = 8.2 Hz, 2H), 7.48 (d, *J* = 8.6 Hz, 2H), 7.09 (s, 1H), 6.97 (d, *J* = 8.4 Hz, 2H), 5.30 (s, 2H), and 3.85 (s, 3H) ppm. ^13^C-NMR (176 MHz, CDCl_3_) δ 161.60, 160.19, 159.57, 153.63, 136.04, 135.50, 130.17, 129.53, 129.23, 128.88, 117.15, 114.22, 110.19, 87.14, and 55.45 ppm. Mass (ESI): *m/z* 336.116 [M(^35^Cl) + H]^+^; 338.0885 [M(^37^Cl) + H]^+^; 358.0559 [M(^35^Cl) + Na]^+^; 359.9924 [M(^37^Cl) + Na]^+^.

### 3.3. Cytotoxicity Assay of APNs ***1***–***6***

The cytotoxic activity of compounds **1**–**6** was evaluated against mammary gland breast cancer (MCF-7) and breast cancer (MDA-MB-231) using the MTT assay as previously described [39,40]. The cell lines were cultured in RPMI-1640 medium with 10% fetal bovine serum. Antibiotics (100 units/mL of penicillin and 100 µg/mL of streptomycin) were added at 37 °C in a 5% CO_2_ incubator. The cell lines were seeded in a 96-well plate at a density of 1.0 × 10^4^ cells/well at 37 °C for 48 h under 5% CO_2_. After incubation, the cells were treated with different concentrations of the compounds and incubated for 24 h. After 24 h of drug treatment, 20 µL of MTT solution at 5mg/mL was added and incubated for 4 h. Dimethyl sulfoxide (DMSO) in a volume of 100 µL was added to each well to dissolve the purple formazan formed. The colorimetric assay was measured and recorded at an absorbance of 570 nm using a plate reader (EXL 800, Winooski, VT, USA). The relative cell viability in percentage was calculated as (A570 of treated samples/A570 of untreated sample) × 100.

### 3.4. Computational Studies of APNs ***1***–***6***

The initial molecular structures of all of the examined structures were drawn using the GaussView 6.0.16 program [41] and optimized at the DFT/b3lyp/6-311++g(d,p) level using Gaussian 16, Revision C.01 [42]. Geometry-optimized structures were subjected to further TD-DFT calculation using Gaussian 16, Revision C.01 by computing 5 singlet excited states on top of the structures obtained at the TD-DFT/b3lyp/6-311++g(d,p) level of theory with the solvent polarizable continuum model (PCM) in toluene, tetrahydrofuran (THF), dichloromethane (DCM), dimethyl sulfoxide (DMSO), and methanol solvents. A vibrational analysis was carried out for each optimized molecule to ensure that they were at a vibrational energy minimum and had no imaginary frequencies. The Frontier Molecular Orbital (FMO) in terms of the energy distribution from HOMO (highest occupied molecular orbital) to LUMO (lowest unoccupied molecular orbital) and a molecular electrostatic potential (MEP) map were calculated using the GaussView 6.0.16 program. The disparity in energy levels between the highest occupied molecular orbital (HOMO) and the lowest unoccupied molecular orbital (LUMO), commonly referred to as the H-L energy gap, correlates with the polarizability of a species and was calculated according to Koopmans’ theorem [43]. Several chemical descriptors, such as energy gap (E_Gap_), ionization potential (IP), electron affinity (EA), chemical potential (μ), electronegativity (χ), hardness (η), softness (σ), and electrophilicity (ω), were calculated using the following equations:

E_Gap_ (eV) = (E_LUMO_ − E_HOMO_); IP (eV) = −E_HOMO_; EA (eV) = −E_LUMO_;

μ (eV) = (IP + EA)/2; χ = − μ; η = (IP − EA)/2; σ = 1/η; ω = μ^2^/2η

where μ and η were used to derive the electrophilicity indexes. 

## 4. Conclusions

In conclusion, this study presents a comprehensive investigation of APNs (**1**–**6**), highlighting their diverse biological activities and potential as fluorescent sensors. The convenient synthesis of **1**–**6** and their evaluation against cancer cell lines reveal compound **3** as a highly potent cytotoxic agent, surpassing the effectiveness of Doxorubicin. The photophysical characterization demonstrates the significant influence of solvent choice and electron-donating/withdrawing substitutions on the fluorescence behavior of **1**–**6**, with compound **3** consistently exhibiting superior fluorescence properties. The TD-DFT analysis reveals comparable HOMO-LUMO energy gaps among the synthesized **1**–**6**, indicating similar electronic delocalization and reactivity patterns. Differences in electronegativity values and dipole moments suggest variations in electron-attracting capabilities and potential electron transfer reactions. These findings provide valuable insights into the molecular-level characteristics, structure–property relationships, and potential applications of **1**–**6** in various fields. Further optimization and development of **1**–**6** could lead to effective therapeutic options and their use as fluorescent sensors for monitoring photopolymerization processes.

## Figures and Tables

**Table 1:** IC_50_ values of the synthesized APNs (**1**–**6**).

Cell Lines	IC_50_ * of APNs (1–6)						
	1	2	3	4	5	6	Dox **
MDA-MB-231	78.28 ± 3.9	8.01 ± 0.5	1.81 ± 0.1	6.93 ± 0.4	15.52 ± 1.2	10.23 ± 0.8	3.18 ± 0.1
MCF-7	>100	16.20 ± 1.3	2.85 ± 0.1	5.59 ± 0.3	20.07 ± 1.5	9.47 ± 0.7	4.17 ± 0.2

* IC_50_ values are the mean ± SD of triplicate measurements; ** standard drug “Doxorubicin”.

**Table 2:** Emission maxima (λ_em_, nm) of synthesized APNs (**1**–**6**) in different solvents.

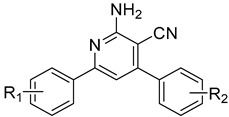	Emission Maxima (λ_em_, nm) at a Conc. of 1.45 × 10^−8^ M				
	DCM	DMSO	MeOH	THF	Toluene
**1**: R_1_ = R_2_ = H	403	425	415	412	401
**2**: R_1_ = 3-OCH_3_; R_2_ = H	401	427	414	412	404
**3**: R_1_ = 3-OCH_3_; R_2_ = 4Cl	416	433	419	418	405
**4**: R_1_ = 3-OCH_3_; R_2_ = 4-OCH_3_	396	420	410	407	397
**5**: R_1_ = 4-OCH_3_; R_2_ = H	401	422	411	410	401
**6**: R_1_ = 4-OCH_3_; R_2_ = 4Cl	404	431	418	417	404

**Table 3:** HOMO-LUMO energies of the synthesized APNs (**1**–**6**) in DMSO and MeOH solvent system *.

Compounds	Solvents	HOMO Energy	LUMO Energy	HOMO-LUMO Gap (ΔE)	Ionization Potential (IP)	Electron Affinity (EA)	Electro Negativity (c)	Chemical Potential (μ)	Hardness (h)	Softness (S)	Electrophilicity Index (ω)	Dipole Moment (Debye)
**1**	DMSO	−6.226	−2.610	3.617	2.226	2.610	4.418	−4.418	1.808	0.553	5.397	5.086
	MeOH	−6.226	−2.608	3.617	6.226	2.608	4.417	−4.417	1.809	0.553	5.393	5.064
**2**	DMSO	−6.194	−2.604	3.590	6.194	2.604	4.399	−4.399	1.795	0.557	5.390	6.854
	MeOH	−6.192	−2.602	3.591	6.192	2.602	4.397	−4.397	1.795	0.557	5.384	6.826
**3**	DMSO	−6.216	−2.655	3.561	6.216	2.655	4.436	−4.436	1.780	0.562	5.526	11.835
	MeOH	−6.216	−2.655	3.561	6.216	2.655	4.435	−4.435	1.781	0.562	5.524	11.787
**4**	DMSO	−6.147	−2.531	3.616	6.147	2.531	4.339	−4.339	1.808	0.553	5.206	5.471
	MeOH	−6.145	−2.528	3.618	6.145	2.528	4.337	−4.337	1.809	0.553	5.198	5.449
**5**	DMSO	−5.981	−2.493	3.488	5.981	2.493	4.237	−4.237	1.744	0.573	5.147	6.761
	MeOH	−5.980	−2.491	3.489	5.980	2.491	4.235	−4.235	1.745	0.573	5.141	6.726
**6**	DMSO	−6.000	−2.558	3.442	6.000	2.558	4.279	−4.279	1.721	0.581	5.321	8.135
	MeOH	−6.001	−2.558	3.443	6.001	2.558	4.279	−4.279	1.722	0.581	5.318	8.098

* Note: HOMO = highest occupied molecular orbital; LUMO = lowest unoccupied molecular orbital; and ΔEgap = ELUMO − EHOMO. Calculations were performed at the RB3LYP TD-SCP/6-311++G(d,p) level.

**Table 4:** TD-DFT-calculated emission maxima (λ_em_, nm) of synthesized APNs (**1**–**6**) in different solvents.

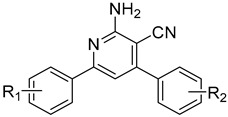	TD-DFT Emission Maxima (λ_em_, nm) Calculated by Using the TD-SCF-B3LYP/6311++G(d,p) Level of Theory in Different Solvent Systems				
	DCM	DMSO	MeOH	THF	Toluene
**1**: R_1_ = R_2_ = H	307, 404	309, 408	304, 408	307, 403	303, 394
**2**: R_1_ = 3-OCH_3_; R_2_ = H	304, 410	306, 410	306, 410	304, 405	300, 396
**3**: R_1_ = 3-OCH_3_; R_2_ = 4Cl	308, 414	309, 414	309, 414	307, 405	304, 401
**4**: R_1_ = 3-OCH_3_; R_2_ = 4-OCH_3_	304, 407	306, 411	305, 411	303, 406	299, 396
**5**: R_1_ = 4-OCH_3_; R_2_ = H	315, 416	316, 420	316, 420	315, 415	311, 404
**6**: R_1_ = 4-OCH_3_; R_2_ = 4Cl	323, 422	325, 427	324, 426	323, 421	320, 412

## Data Availability

The data presented in this study are available in article and Supplementary Materials.

## Supplementary Materials

The following supporting information can be downloaded at https://www.mdpi.com/article/10.3390/molecules29081808/s1, ^1^H-NMR, ^13^C-NMR, and mass spectra of compounds **1**–**6**. TD-DFT calculation raw files.
