# Supplementary material for: Synthesis, Cytotoxicity, and Photophysical Investigations of 2-Amino-4,6-diphenylnicotinonitriles: An Experimental and Theoretical Study
Source: Molecules. 2024 Apr 16;29(8):1808. doi: 10.3390/molecules29081808 (PMC11055175; doi:10.3390/molecules29081808)

Supporting data

Synthesis, Cytotoxicity and Photophysical Investigations of 2-Amino-4,6-diphenylnicotinonitriles: An Experimental and Theoretical Study

Alwah R. Al-Ghamdi,[1] Shofiur Rahman,[2] Reem I. Al-Wabli,[1] Maha S. Al-Mutairi[1] and A. F. M. Motiur Rahman[1,*]

1 Department of Pharmaceutical Chemistry, College of Pharmacy, King Saud University, Riyadh 11451, Saudi Arabia; 439204477@student.ksu.edu.sa (A.R.A-G.), ralwabli@ksu.edu.sa (R.I.A.-W.), malmutbiri@ksu.edu.sa (M.S.A-M.)

2 College of Science, King Saud University, Riyadh 11451, Saudi Arabia mrahman1@ksu.edu.sa (S.A.),

*Correspondence: afmrahman@ksu.edu.sa; Tel.: +966-11-46-70237 (A.F.M.M.R.)

Content	Page No.	Content	Page No.
^{1}H-NMR of **1**	3	^{1}H-NMR of **4**	12
^{13}C-NMR of **1**	4	^{13}C-NMR of **4**	13
Mass Spectra of **1**	5	Mass Spectra of **4**	14
^{1}H-NMR of **2**	6	^{1}H-NMR of **5**	15
^{13}C-NMR of **2**	7	^{13}C-NMR of **5**	16
Mass Spectra of **2**	8	Mass Spectra of **5**	17
^{1}H-NMR of **3**	9	^{1}H-NMR of **6**	18
^{13}C-NMR of **3**	10	^{13}C-NMR of **6**	19
Mass Spectra of **3**	11	Mass Spectra of **6**	20

^{1}H-NMR of **1**

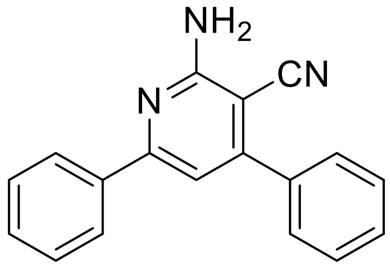

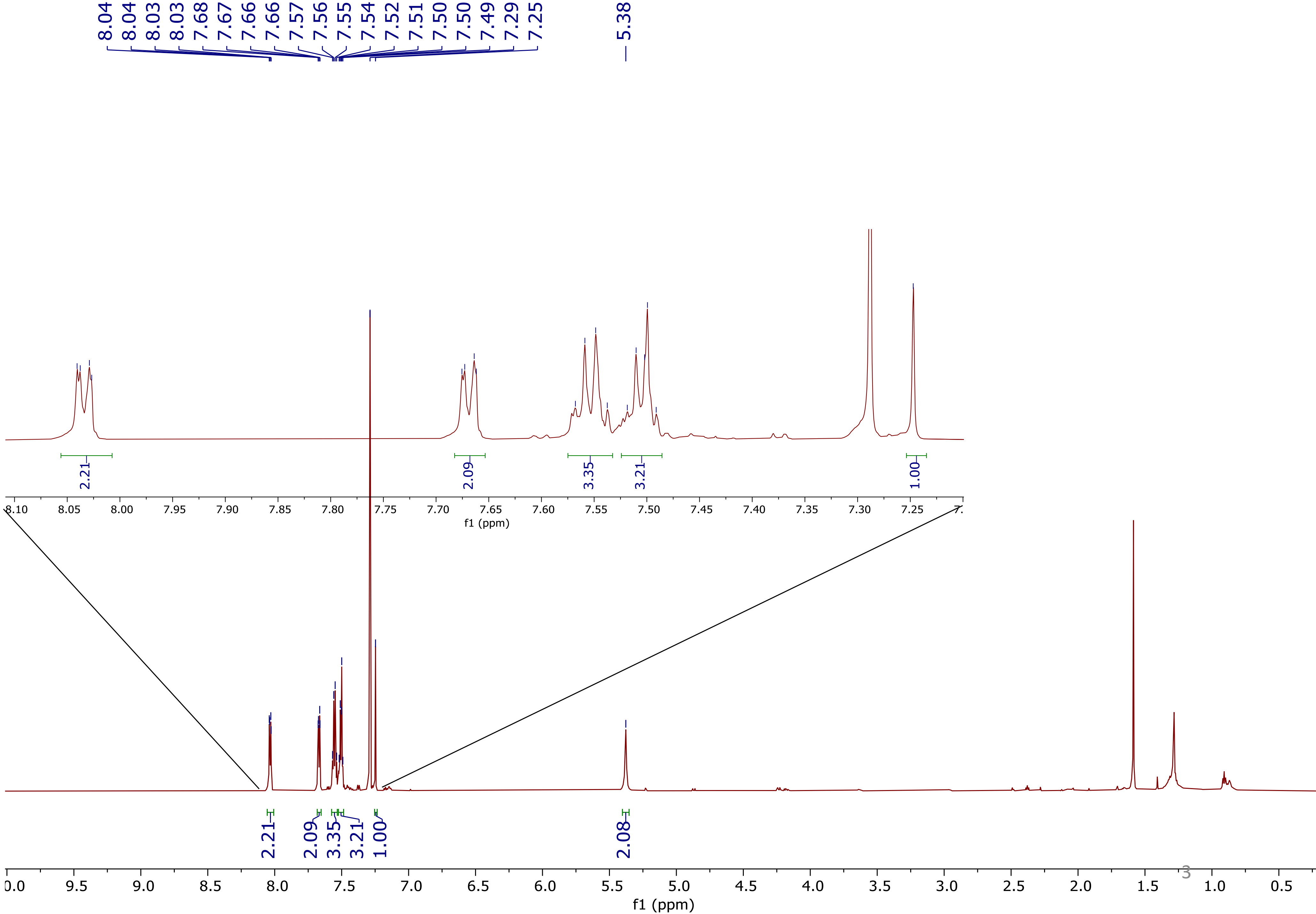
8.04
8.04
8.03
8.03
7.68
7.67
7.66
7.66
7.57
7.56
7.55
7.54
7.52
7.51
7.50
7.50
7.49
7.29
7.25
5.38
2.21
2.09
3.35
3.21
1.00
2.08
f1 (ppm)

^{13}C-NMR of **1**

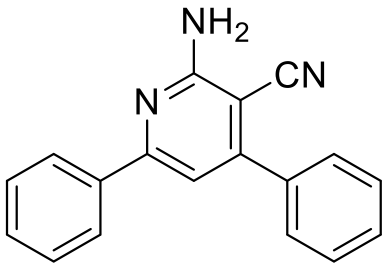

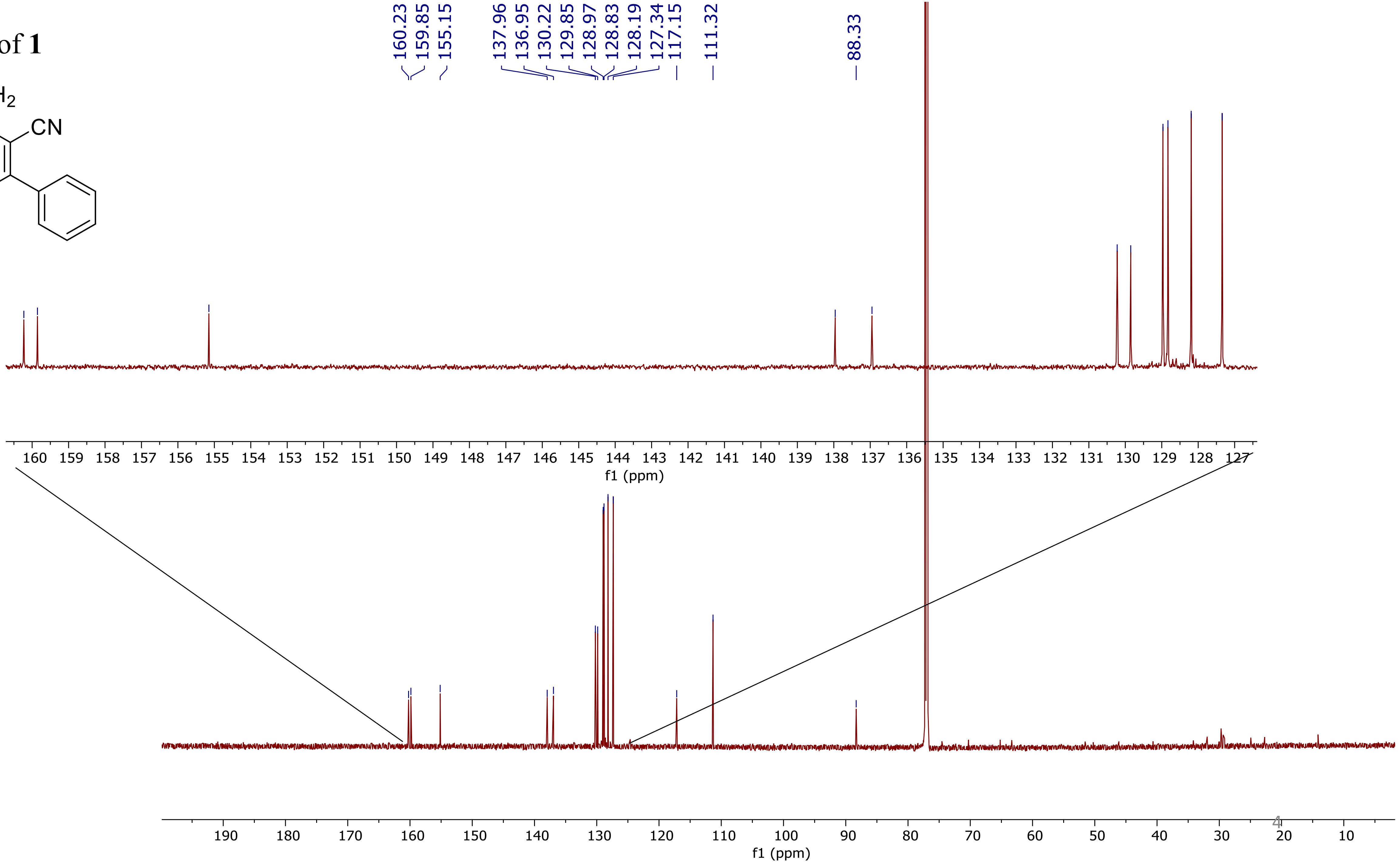
160.23
159.85
155.15
137.96
136.95
130.22
129.85
128.97
128.83
128.19
127.34
117.15
111.32
88.33
f1 (ppm)
f1 (ppm)

Mass Spectra of **1**

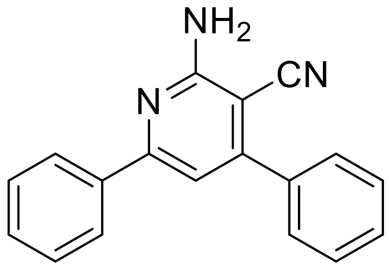

Exact Mass = 271.1109

m/z = 271.9693 $[M+H]^+$; 293.9424 $[M+Na]^+$

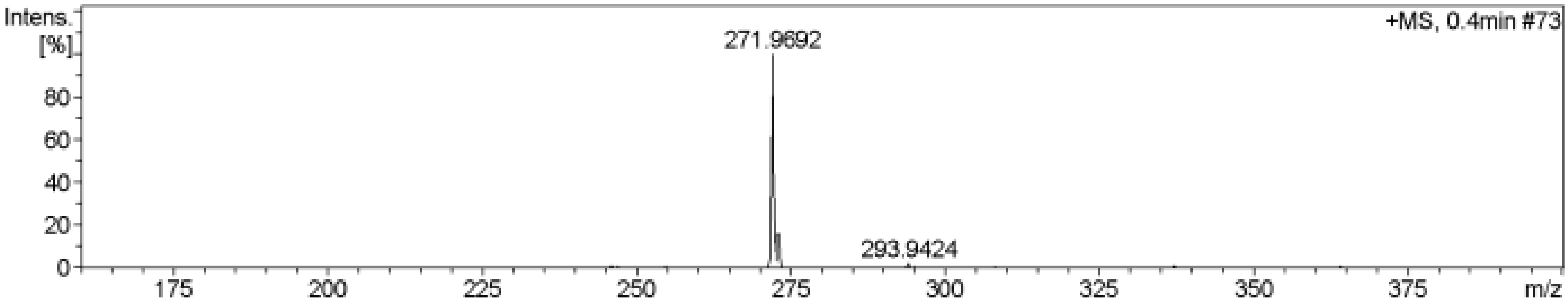

^{1}H-NMR of **2**

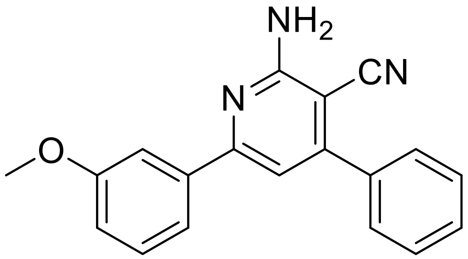
NH2
CN
N
O

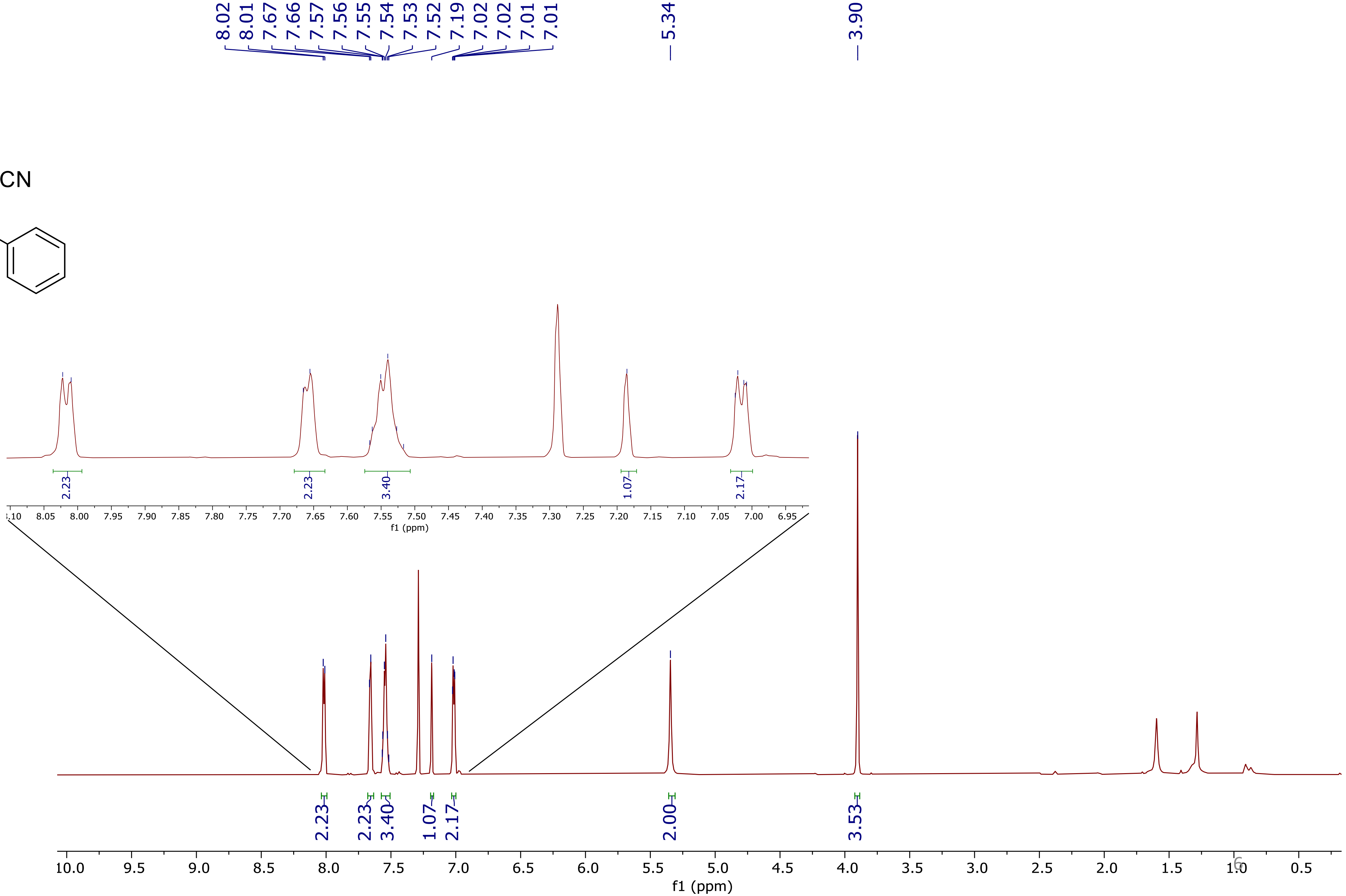
8.02 8.01 7.67 7.66 7.57 7.56 7.55 7.54 7.53 7.52 7.19 7.02 7.02 7.01 7.01
5.34
3.90
2.23
2.23
3.40
1.07
2.17
2.00
3.53
f1 (ppm)
f1 (ppm)

^{13}C-NMR of **2**

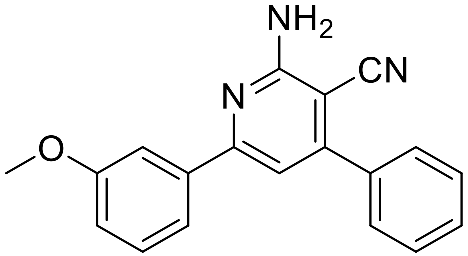
NH2
CN
N
O

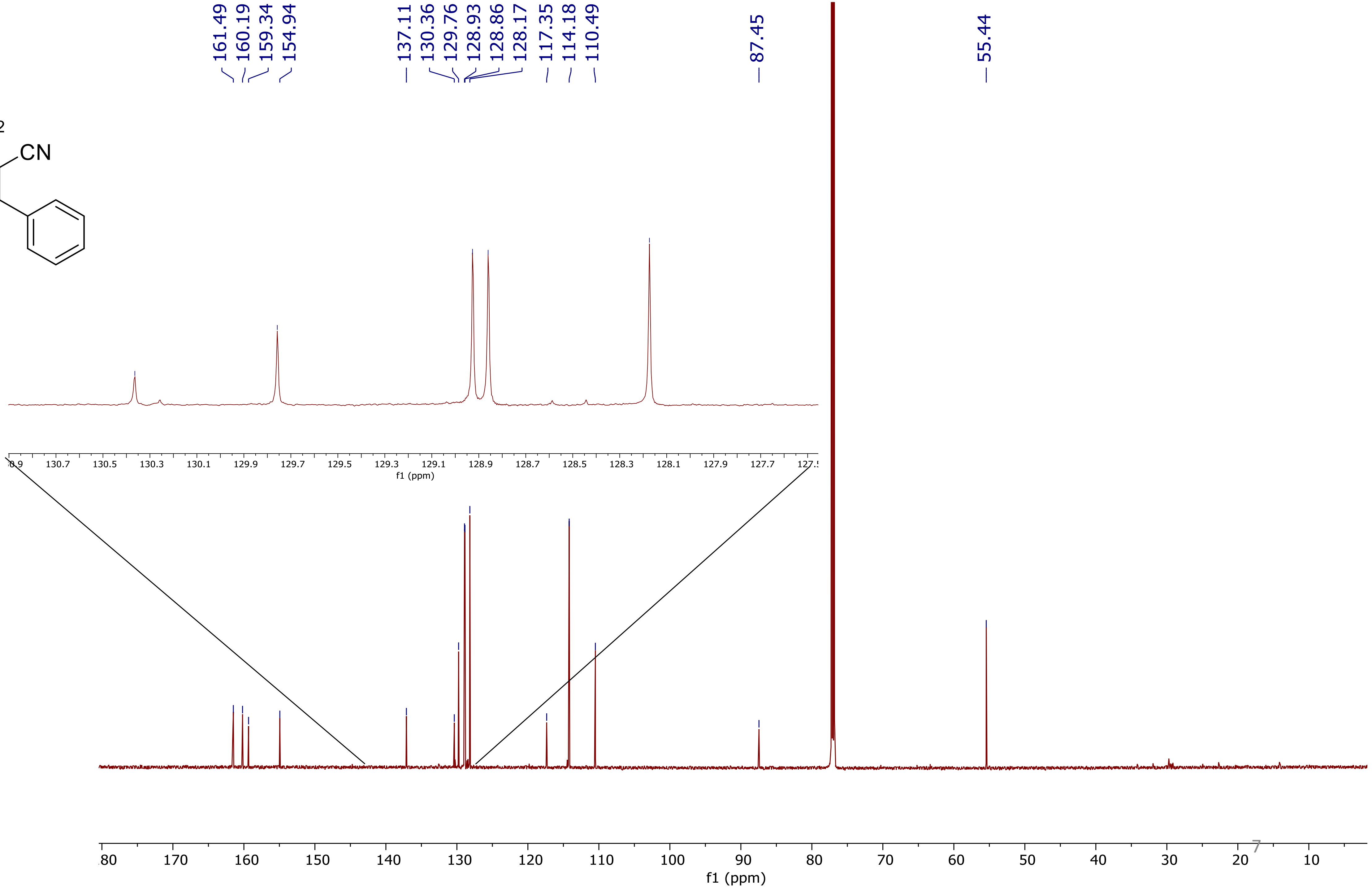
161.49
160.19
159.34
154.94
137.11
130.36
129.76
128.93
128.86
128.17
117.35
114.18
110.49
87.45
55.44
f1 (ppm)
f1 (ppm)

Mass Spectra of **2**

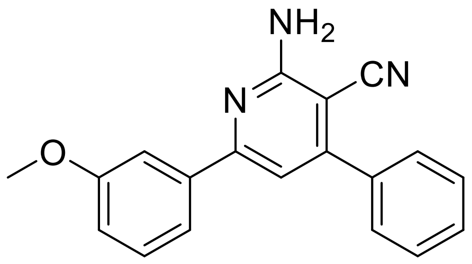

Exact Mass = 301.1215

m/z = 302.0671 $[M+H]^+$; 324.0705 $[M+Na]^+$

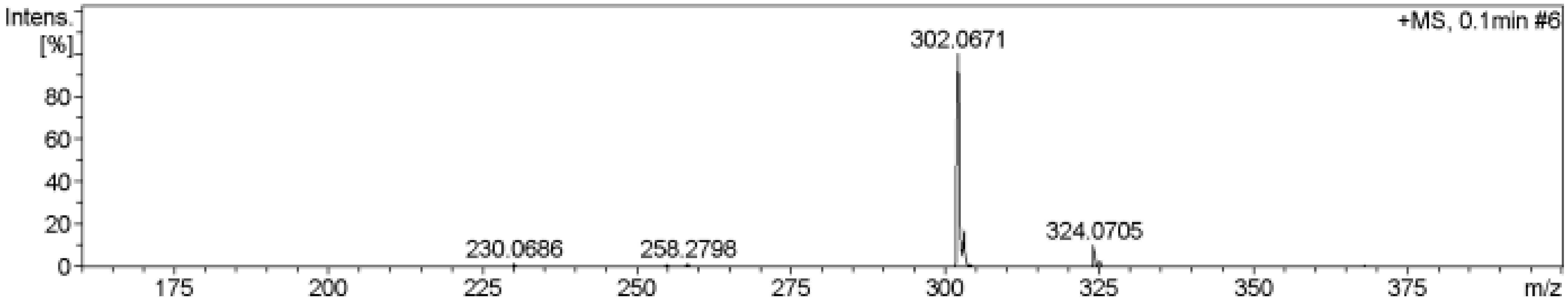

^{1}H-NMR of **3**

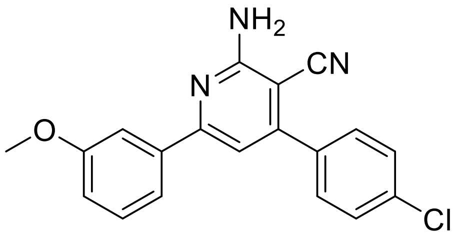
NH2
CN
N
O
Cl

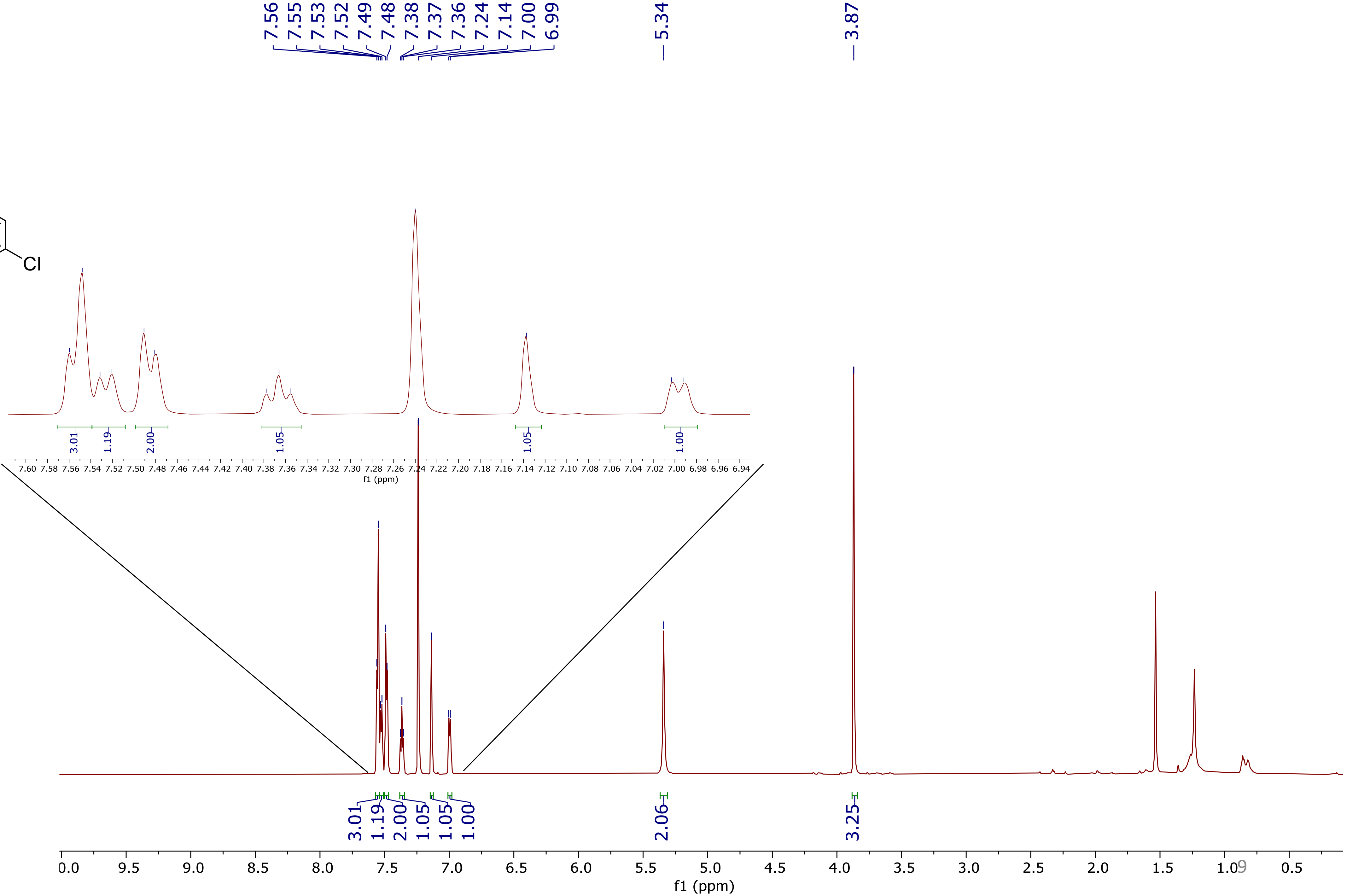
7.56
7.55
7.53
7.52
7.49
7.48
7.38
7.37
7.36
7.24
7.14
7.00
6.99
5.34
3.87
3.01
1.19
2.00
1.05
1.05
1.00
2.06
3.25
f1 (ppm)

^{13}C-NMR of **3**

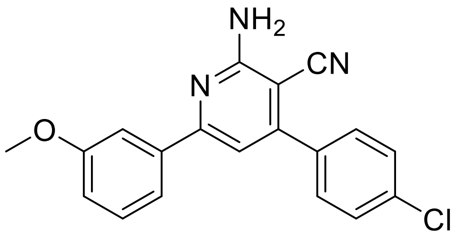
NH2
CN
N
O
Cl

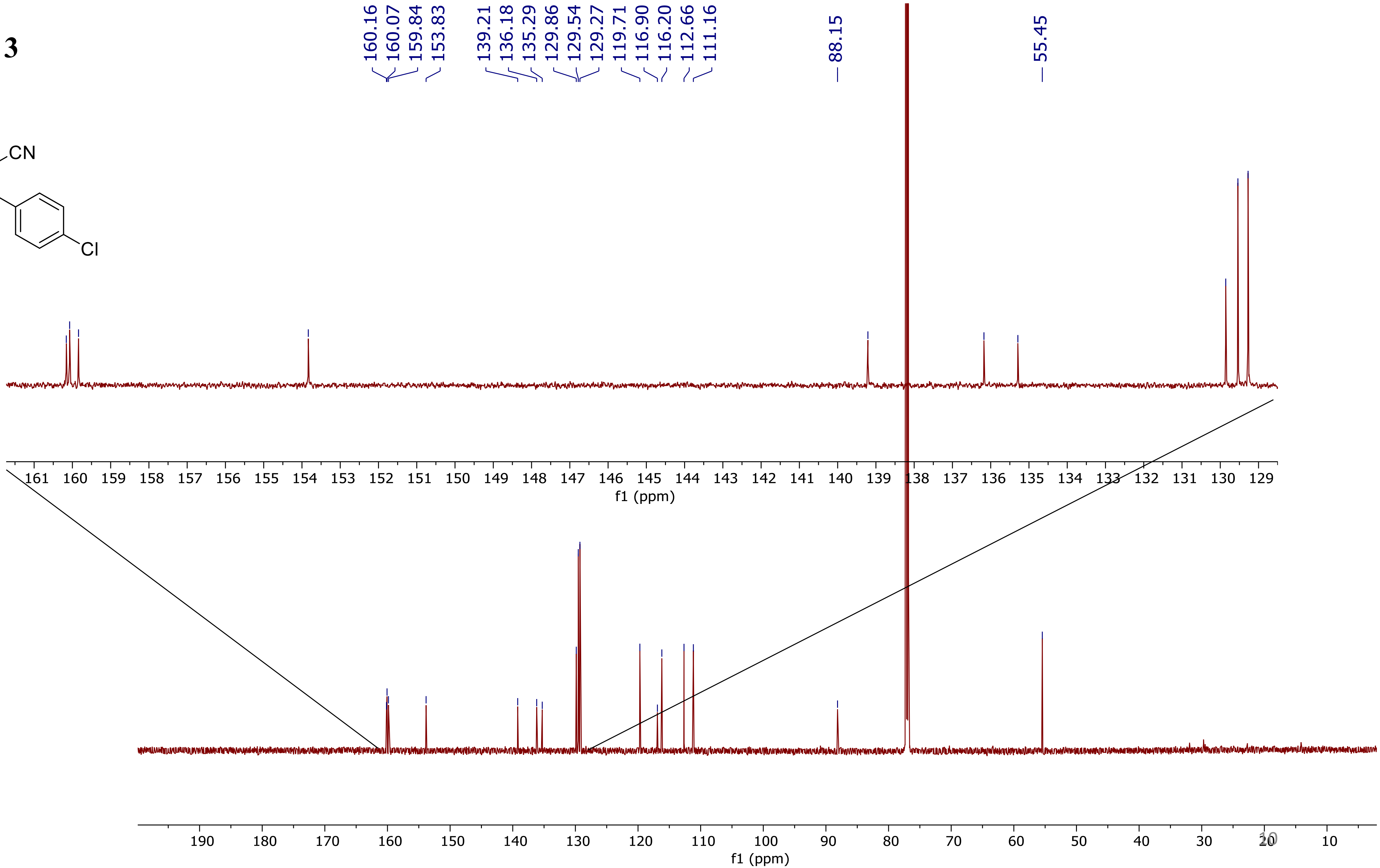
160.16
160.07
159.84
153.83
139.21
136.18
135.29
129.86
129.54
129.27
119.71
116.90
116.20
112.66
111.16
88.15
55.45
161 160 159 158 157 156 155 154 153 152 151 150 149 148 147 146 145 144 143 142 141 140 139 138 137 136 135 134 133 132 131 130 129
f1 (ppm)
190 180 170 160 150 140 130 120 110 100 90 80 70 60 50 40 30 20 10
f1 (ppm)

Mass Spectra of **3**

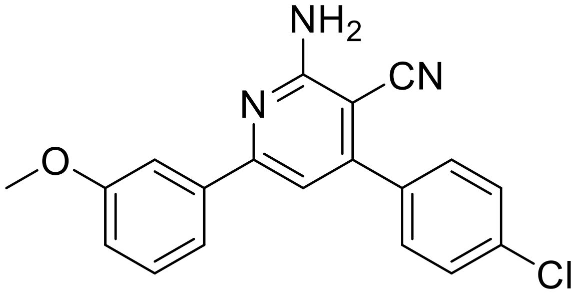

Exact Mass = 335.0825

m/z 336.0031 [M(^{35}Cl)+H]$^+$; 338.0375 [M(^{37}Cl)+H]$^+$.

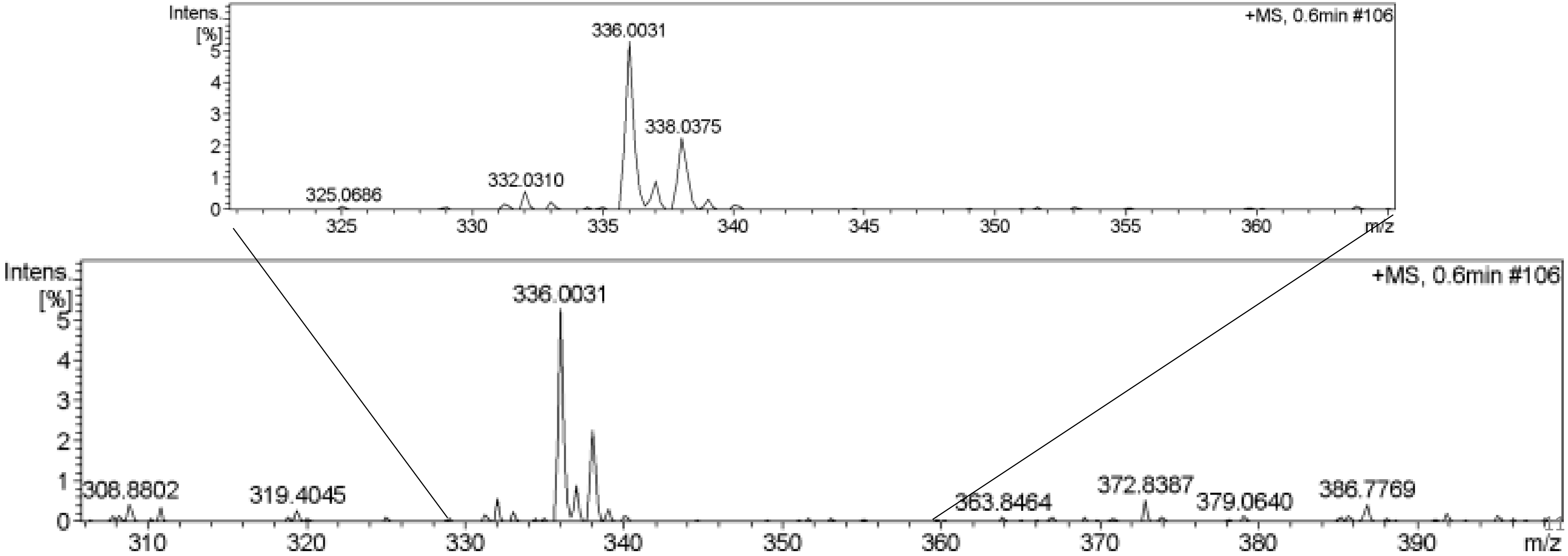

^{1}H-NMR of **4**

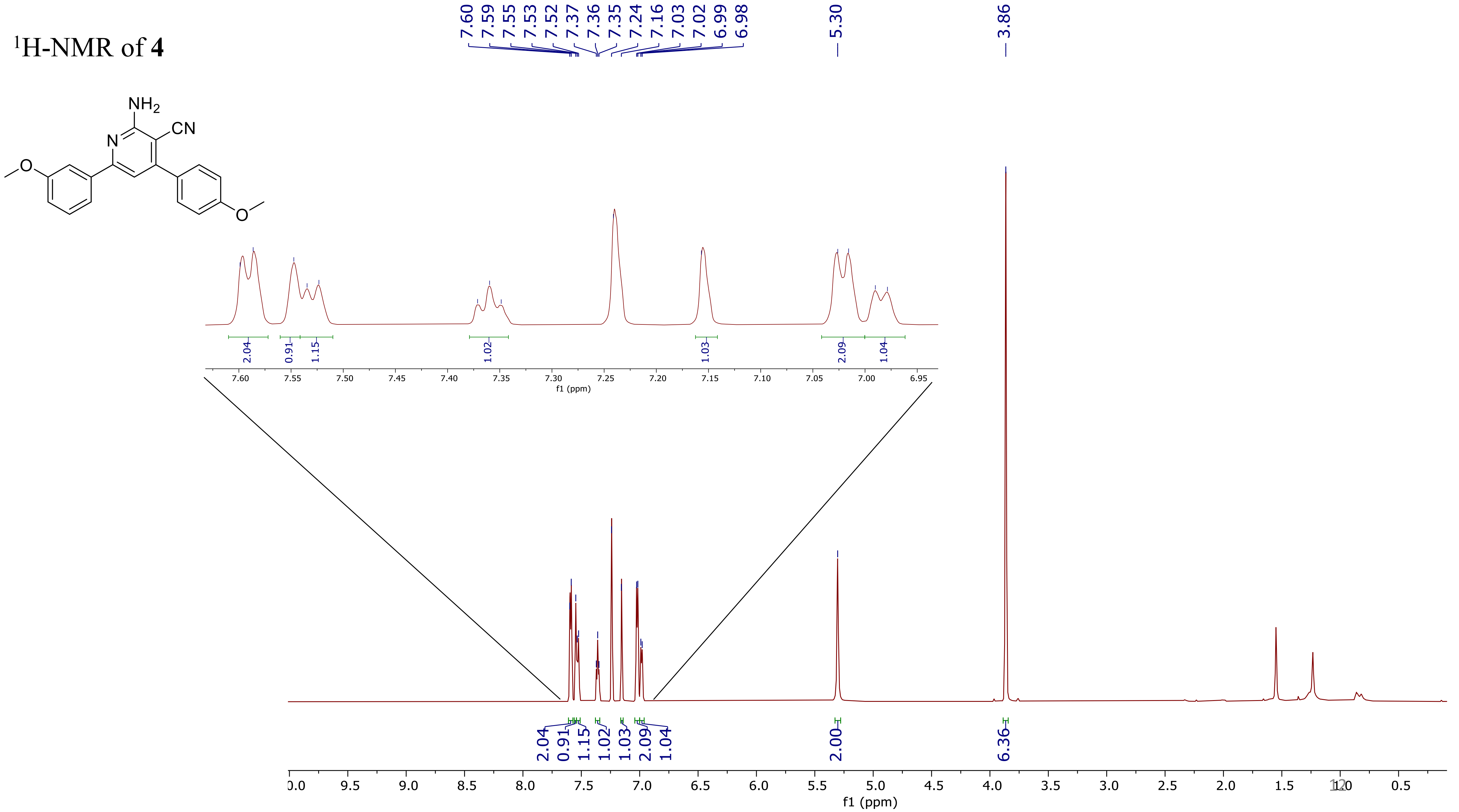

NH2
CN
N
O
O
7.60 7.59 7.55 7.53 7.52 7.37 7.36 7.35 7.24 7.16 7.03 7.02 6.99 6.98
5.30
3.86
2.04 0.91 1.15 1.02 1.03 2.09 1.04
2.00
6.36
f1 (ppm)

^{13}C-NMR of **4**

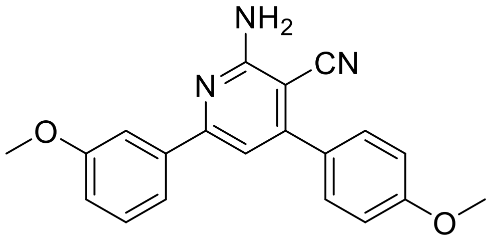
NH2
CN
N
O
O

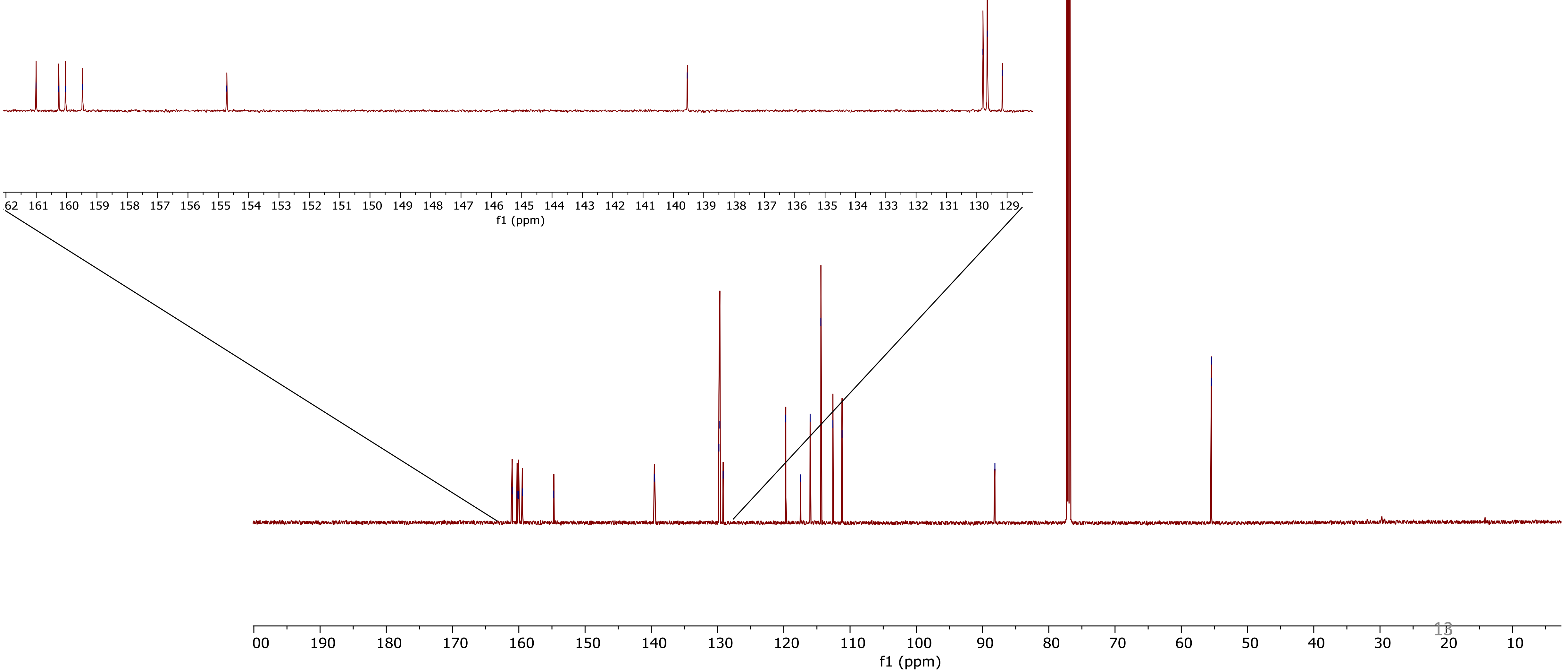
161.01
160.26
160.04
159.48
154.72
139.55
129.80
129.65
129.16
119.71
117.47
116.03
114.40
112.59
111.21
88.14
55.45
55.44
f1 (ppm)
f1 (ppm)

Mass Spectra of **4**

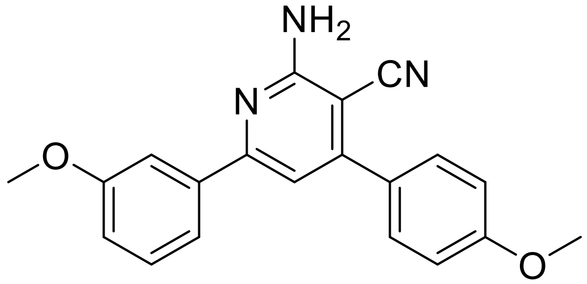

Exact Mass = 331.1321

m/z = 332.1401 [M+H]$^+$; 354.0757 [M+Na]$^+$; 370.0165 [M+K]$^+$;

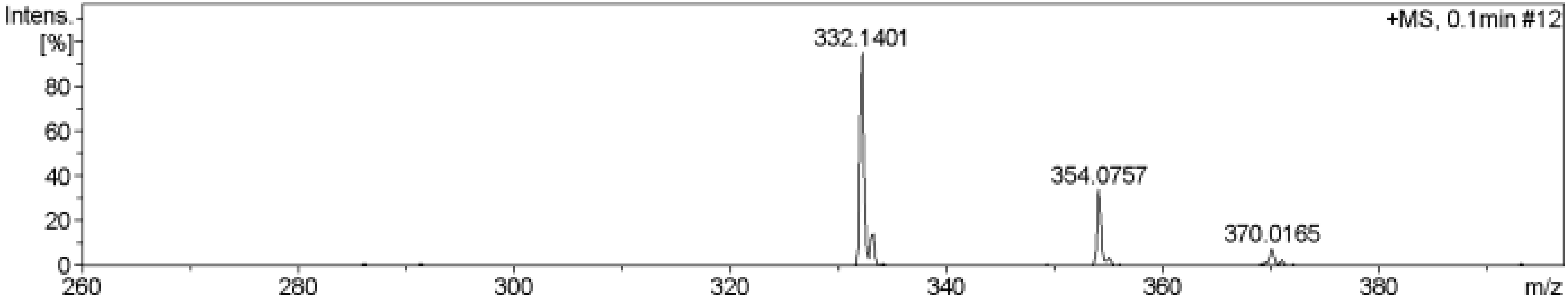

^{1}H-NMR of **5**

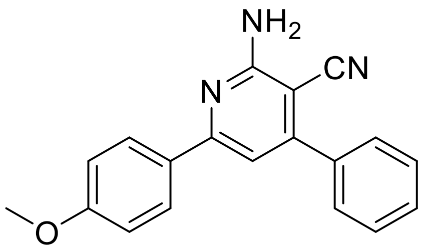
NH2
CN
N
O

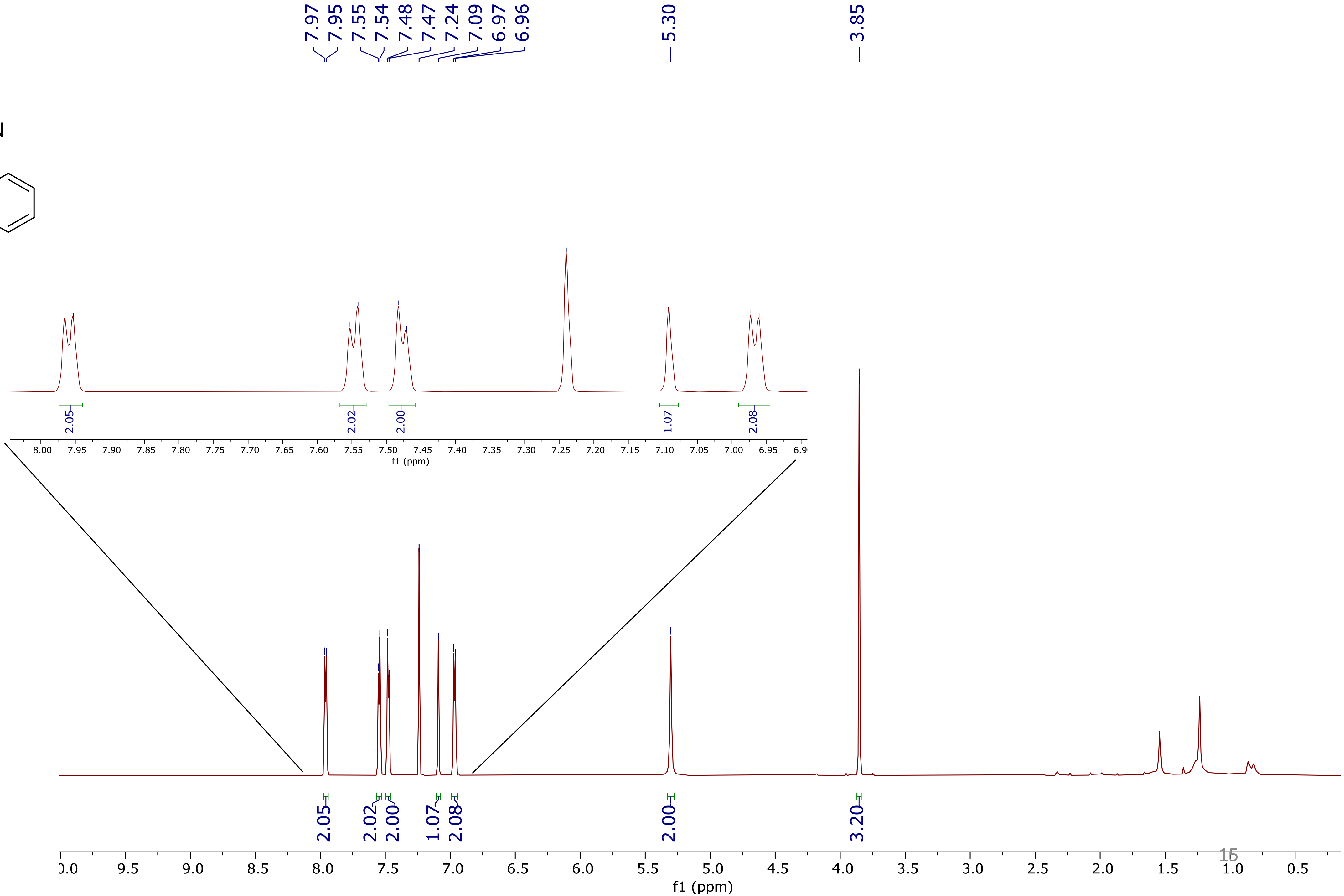
7.97
7.95
7.55
7.54
7.48
7.47
7.24
7.09
6.97
6.96
5.30
3.85
2.05
2.02
2.00
1.07
2.08
2.00
3.20
f1 (ppm)

^{13}C-NMR of **5**

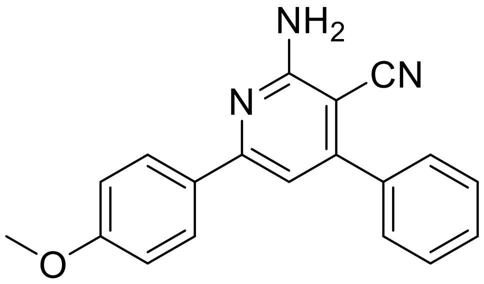
NH2
CN
N
O

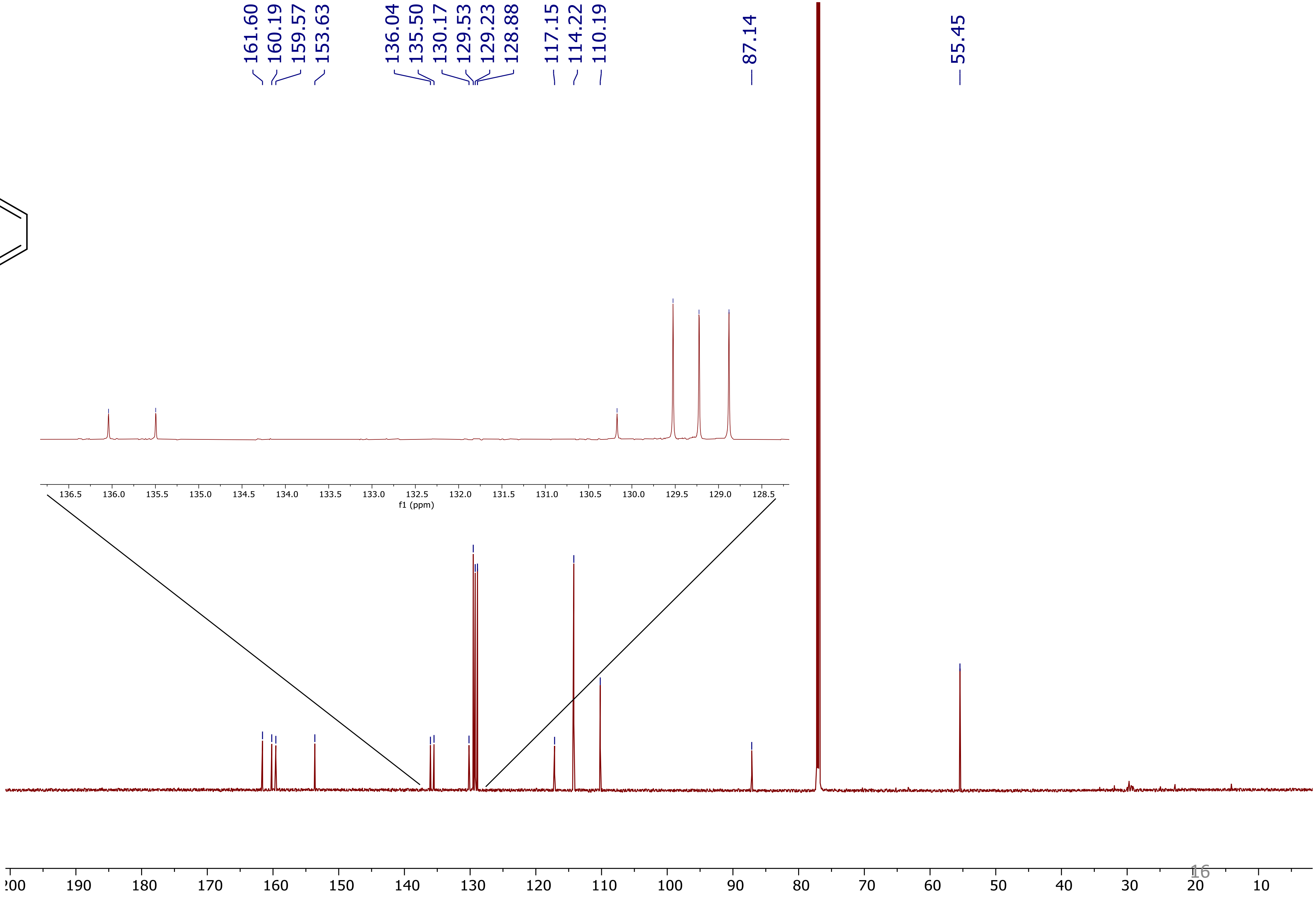
161.60
160.19
159.57
153.63
136.04
135.50
130.17
129.53
129.23
128.88
117.15
114.22
110.19
87.14
55.45
f1 (ppm)
f1 (ppm)

Mass Spectra of **5**

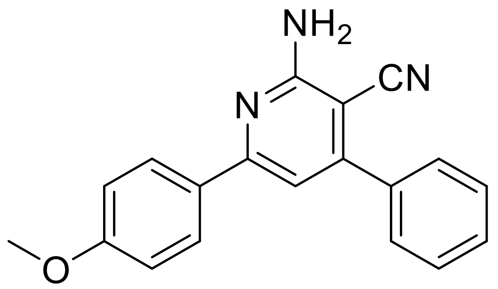

Exact Mass = 301.1215
m/z = 302.0650 [M+H]$^+$; 324.0657 [M+Na]$^+$

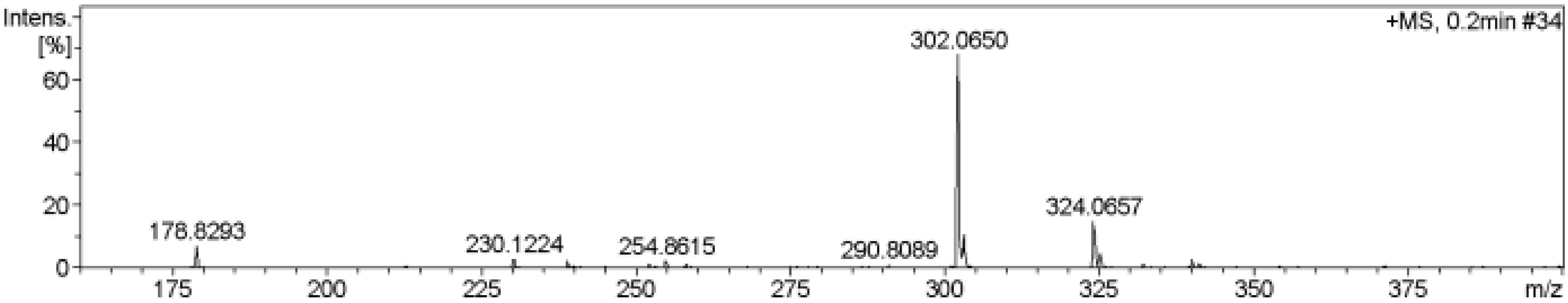

^{1}H-NMR of **6**

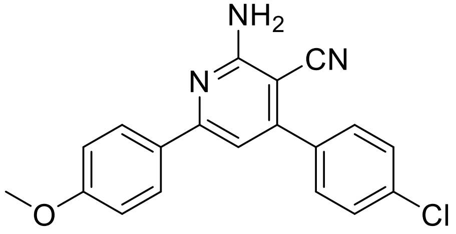
NH2
CN
N
O
Cl

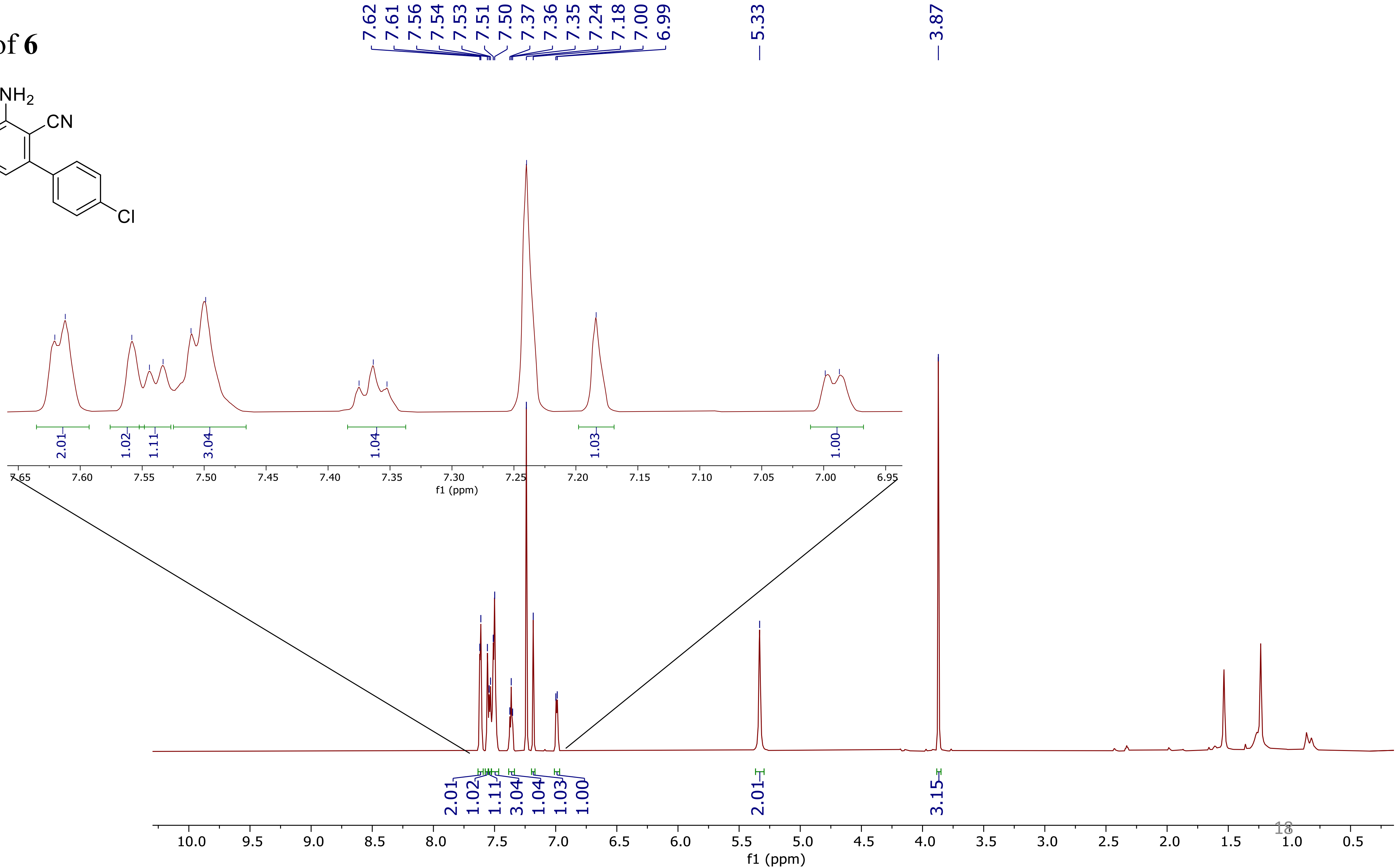
7.62
7.61
7.56
7.54
7.53
7.51
7.50
7.37
7.36
7.35
7.24
7.18
7.00
6.99
5.33
3.87
2.01
1.02
1.11
3.04
1.04
1.03
1.00
2.01
3.15
f1 (ppm)
f1 (ppm)

^{13}C-NMR of **6**

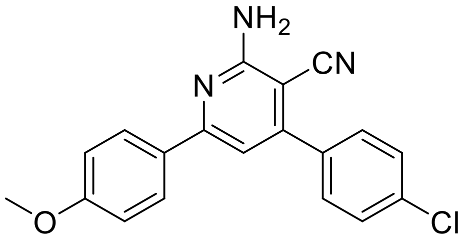
NH2
CN
N
O
Cl

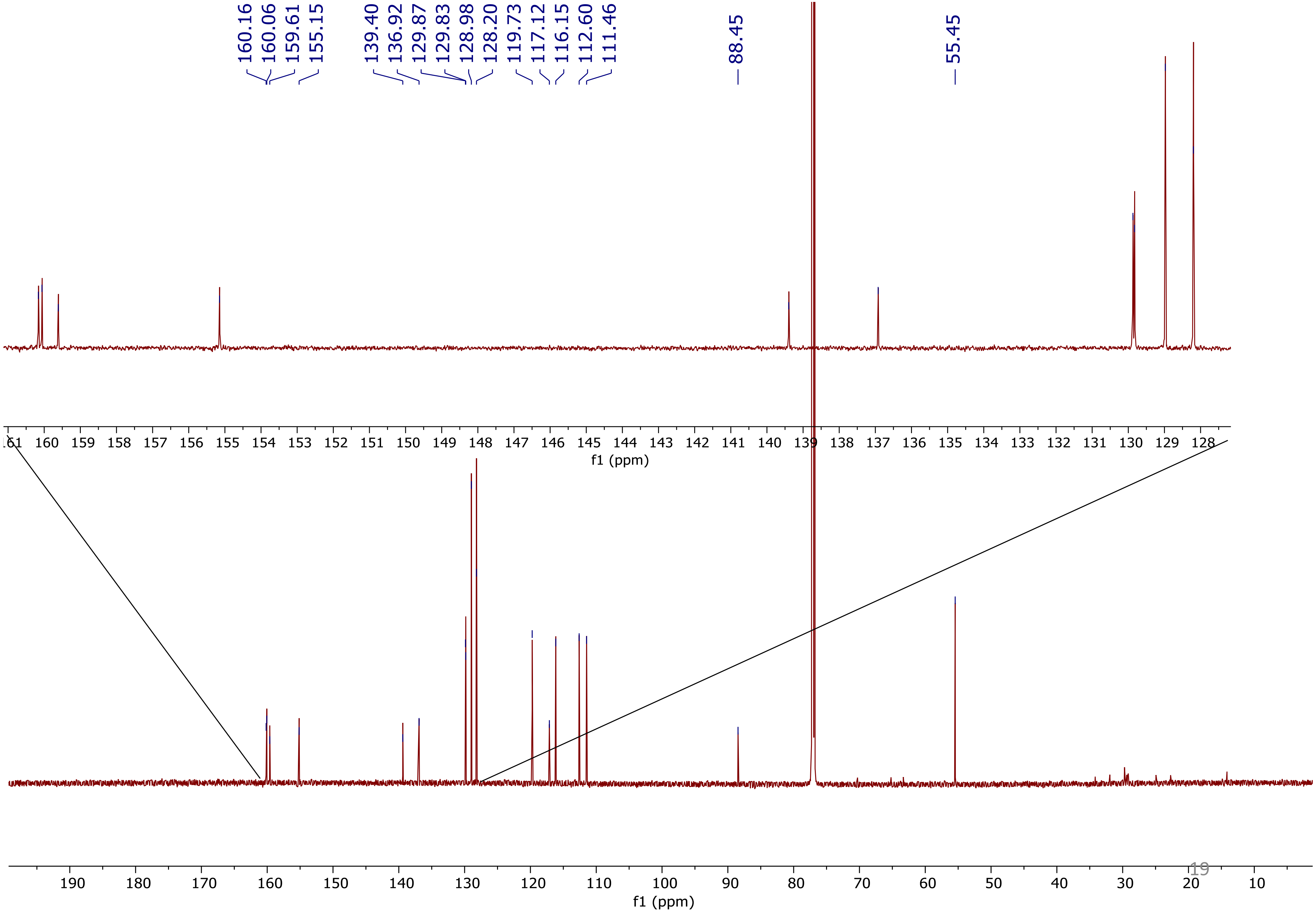
160.16
160.06
159.61
155.15
139.40
136.92
129.87
129.83
128.98
128.20
119.73
117.12
116.15
112.60
111.46
88.45
55.45
f1 (ppm)
f1 (ppm)

Mass Spectra of **6**

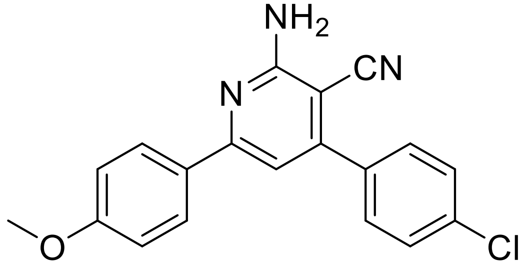

Exact Mass = 335.0825

m/z 336.116 [M(^{35}Cl)+H]$^+$; 338.0885 [M(^{37}Cl)+H]$^+$; 358.0559 [M(^{35}Cl)+Na]$^+$; 359.9924 [M(^{37}Cl)+Na]$^+$.

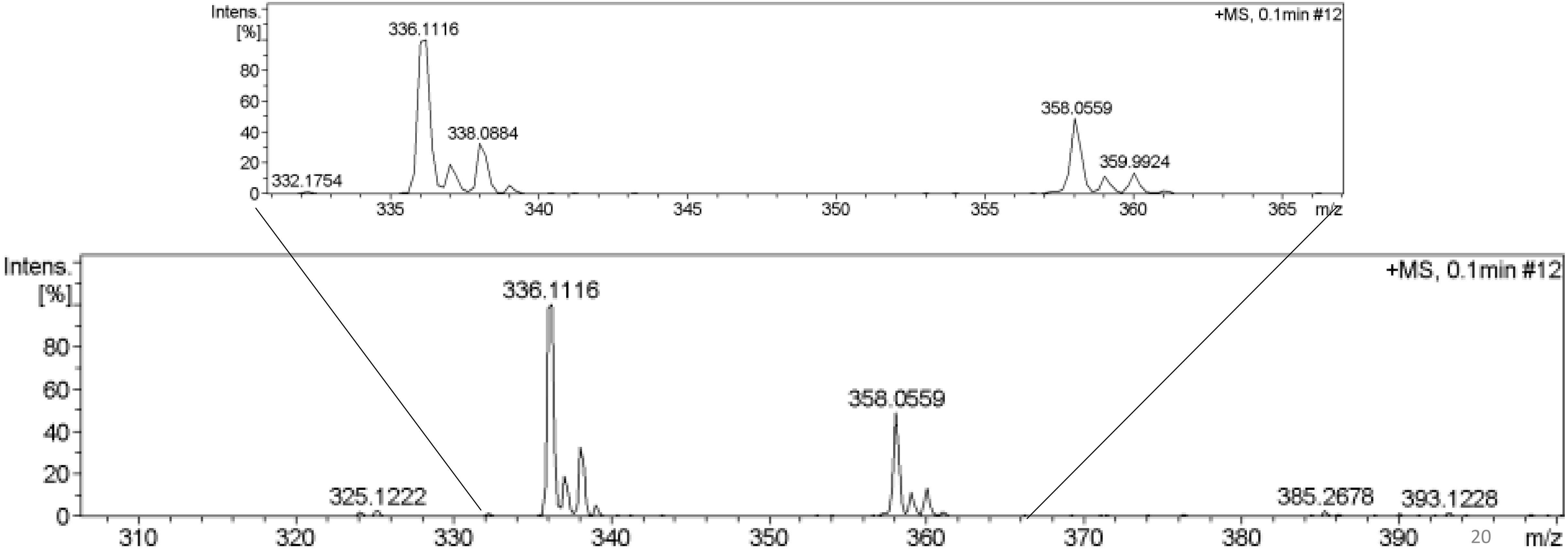

Supplement: Supplementary file 1 [file molecules-29-01808-s001.zip › molecules-2934970-supplementary.pdf]
